# Changes in Quadriceps Force Control and Torque Quality Following Anterior Cruciate Ligament Injury and Reconstruction: Associations with Functional Performance—A Systematic Review and Meta-Analysis

**DOI:** 10.1186/s40798-026-00999-x

**Published:** 2026-05-11

**Authors:** Fatemeh Azadinia, Fatemeh Shamsi, Ismail Ebrahimi-Takamjani, Omid Rasouli

**Affiliations:** 1 Rehabilitation Research Center, Department of Orthotics and Prosthetics, School of Rehabilitation Sciences, Iran University of Medical Sciences, Tehran, Iran; 2 Department of Neuroscience, School of Advanced Medical Sciences and Technologies, Shiraz University of Medical Sciences, Shiraz, Iran; 3 Rehabilitation Research Center, Department of Physiotherapy, School of Rehabilitation Sciences, Iran University of Medical Sciences, Tehran, Iran; 4 Department of Rehabilitation Science and Health Technology, Faculty of Health Sciences, Oslo Metropolitan University (OsloMet), Pilestredet 44, Oslo 0167, Norway

**Keywords:** Anterior cruciate ligament, Force control, Force fluctuations, Quadriceps muscle, Knee joint

## Abstract

**Background:**

Consistent force output is a critical indicator of the neuromuscular system’s effectiveness. Although force signals inherently fluctuate, the ability of skeletal muscles to generate accurate and steady force offers insights into the system’s adaptability and its ability to adjust motor control strategies to meet task demands. This systematic review and meta-analysis aimed to synthesize evidence on quadriceps force control in individuals with anterior cruciate ligament (ACL) injury and/or surgical reconstruction (ACLR). Additionally, it sought to explore the relationship between force control measures and physical function outcomes.

**Methods:**

A literature search was conducted across several databases, including PubMed, EMBASE, Scopus, Web of Science, and SPORTDiscus. Risk of bias was assessed using the adapted Newcastle-Ottawa tool. The study included individuals with unilateral ACL injury and/or ACLR, with comparisons to uninjured controls or unaffected contralateral limbs. Primary outcomes included torque quality, force accuracy, and force/torque steadiness, while secondary outcomes included function-related clinical questionnaires and performance tests. Eligible studies consisted of observational studies and baseline data from interventional studies published in English. Standardized mean differences (SMDs) were calculated using a random effects meta-analysis.

**Results:**

A total of 33 studies were included, comprising 20 individuals with ACLR, 12 with ACL injuries, and one with both. The meta-analysis showed significant effects of ACL injury (SMD = 0.84) and ACLR (SMD = 1.57) on quadriceps torque frequency content. Individuals with ACLR had a greater root mean squared error (RMSE) or absolute error (AE) in force output compared to healthy controls (SMD = 0.35) and exhibited a significant difference in the coefficient of variation (CoV) of the force signal (SMD = 0.22), indicating impaired force control in those with ACLR.

**Conclusions:**

ACL injury impairs quadriceps force control in the injured limb, as shown by torque frequency content analysis. While ACL reconstruction is the gold standard for joint stability, it may not fully restore neuromuscular function, potentially compromising physical functioning. However, the limited number of high-quality studies may weaken these conclusions.

**Registration:**

The review protocol was prospectively registered in the International Prospective Register of Systematic Reviews (PROSPERO; registration number: CRD42024571495).

**Supplementary Information:**

The online version contains supplementary material available at 10.1186/s40798-026-00999-x.

## Key Points

- Traditional linear metrics (like CoV and RMSE) are insufficient; nonlinear analyses are more effective in detecting persistent force control impairments following ACL injury and reconstruction.
- Using the contralateral limb as a “normal” benchmark is flawed. The reconstructed limb may appear similar, but shows significant deficits compared to healthy controls, challenging a key assumption in clinical assessments.
- Impaired quadriceps force steadiness is linked to poorer performance on functional tests, highlighting the clinical importance of these neuromuscular deficits.

**Supplementary Information:**

The online version contains supplementary material available at 10.1186/s40798-026-00999-x.

## Background

The anterior cruciate ligament (ACL), a structure that maintains knee joint integrity [1], is frequently injured in active adults [2]. An increased risk of re-injury and decreased physical activity following ACL injury and/or reconstruction may result from compromised functional joint stability [3]. Although abnormal knee kinematics, such as increased anterior tibial translation and joint laxity [4], can lead to joint instability [5]. Functional joint instability appears to be caused by impaired sensorimotor system components, including sensory inputs, motor output, and central processing [6–8]. Although ACL reconstruction (ACLR) surgery and postoperative rehabilitation can restore the joint’s mechanical function, sensorimotor control deficits may last for months or years [9, 10].

Damage to mechanoreceptors in the affected area disrupts afferent input, which can impair proprioception [11]. As a result, inaccurate sensory feedback is transmitted to the central nervous system (CNS), ultimately reducing the ability to generate precise muscle force [12]. Furthermore, impaired sensory input or altered processing of proprioceptive input can affect efferent commands [13]. Impairment of the motor component of the sensorimotor system may also be associated with these disruptions [14]. It may present as altered muscle activation patterns [15], a slower rate of force development in the quadriceps [16], and reduced quadriceps strength [17]. These altered muscle activation strategies may threaten both cartilage health and joint stability. Abnormal joint loading arising from these strategies could potentially increase the risk of early-onset post-traumatic knee osteoarthritis by compromising cartilage health [18]. Additionally, the ability to effectively regulate muscle force may be considerably impaired, leading to knee joint instability [13]. This is why the ability to generate and maintain a consistent force output is considered a key indicator of the neuromuscular system’s effectiveness [19].

Force signals naturally fluctuate, but the ability of skeletal muscles to generate an accurate and steady force, particularly during submaximal voluntary contractions, provides valuable insights into the adaptability of motor control strategies to meet task demands [19]. Understanding the importance of quadriceps force control is essential, as it not only plays a role in counteracting mechanical perturbations to maintain joint loading and health [20] but is also linked to functional performance [21]. The quadriceps muscle plays a significant role in maintaining functional knee stability and lower extremity locomotion. This often leads to routine dynamometry assessment [22], particularly in clinical evaluations of an athlete’s readiness to return to sport. Current rehabilitation guidelines emphasize restoring muscle strength [23, 24]. However, evaluating only the maximum force-generating capacity, which merely represents the peak point on the torque-time curve, overlooks crucial information about muscle force quality and the underlying neuromuscular control mechanisms [19, 25].

Several studies have reported deficits in quadriceps force control in individuals with ACL injury and/or ACLR [26]. Two recent systematic reviews and meta-analyses have concluded impaired muscle force control in people with peripheral musculoskeletal conditions, as well as those with ACL deficiency and/or ACLR [12, 27]. However, some recent studies, which were not included in those reviews, have reported inconsistent findings [28–34]. These inconclusive results may partly reflect heterogeneity from pooling varied outcome measures and contraction types (isometric versus isokinetic). Therefore, conducting an updated meta-analysis that includes recent studies and also performing subgroup analyses based on more relevant outcome measures may help clarify these inconsistencies. Additionally, those reviews have not explored the relationship between the ability to generate and maintain consistent force and physical function.

To address the mentioned limitations, this systematic review and meta-analysis aimed to synthesize existing evidence on quadriceps force control in individuals with ACL injury and/or ACLR. The secondary objective was to explore associations between force control measures and physical function outcomes, including performance-based tests and self-reported knee function.

## Methods

The findings of this review are reported in accordance with the Preferred Reporting Items for Systematic Reviews and Meta-Analyses (PRISMA) statement. The review protocol was prospectively registered in the International Prospective Register of Systematic Reviews (PROSPERO; registration number: CRD42024571495).

### Information Source and Search

A comprehensive search was initially performed in the electronic databases PubMed, EMBASE, Scopus, Web of Science, and SPORTDiscus covering publications from inception up to July 29, 2024. An updated search was conducted on July 28, 2025, to identify newly published studies. The appropriate Medical Subject Headings (MeSH), Emtree terms, and free-text terms related to the patient (P) and outcome (O) components of the PECO framework were combined using the AND operator. No time, language, or design constraints were applied. We customized the search strategy to each database configuration. In addition, the reference lists of the included articles were manually reviewed to identify additional eligible studies.

All retrieved studies were imported into EndNote software. After removing duplicates, two reviewers (FA and FS) independently screened the titles and abstracts. The full texts of potentially eligible studies were then evaluated against the eligibility criteria by the same two reviewers. Disagreements regarding study eligibility were resolved through consensus, with a third reviewer (OR) intervening when needed. Details of the database search were provided in the supplementary materials (Supplementary 1).

### Study Inclusion and Exclusion Criteria

The PECOS framework (Population, Exposure, Comparator, Outcomes, and Study Design) was used to establish inclusion criteria. Individuals with primary, unilateral ACL injury and/or any surgical ACLR were included in the study. Studies with participants who had ACL revision surgery, bilateral injury and/or reconstruction, or post-traumatic knee osteoarthritis were excluded. Comparisons were made with an uninjured control group, defined as individuals who had never had a knee injury or surgery, or within-subject comparisons with the unaffected contralateral limb.

The primary outcome included linear or non-linear measures of torque quality, force accuracy, and force/torque steadiness during isometric, concentric, and eccentric contractions. In addition, the secondary outcomes were function-related clinical questionnaires and/or functional performance tests. All observational studies (including cohort, cross-sectional, and case-control designs) and baseline data from prospective interventional studies were eligible. These studies included if baseline parameters related to force control or torque quality were compared to the unaffected contralateral limb or healthy controls. All studies published in English in peer-reviewed scientific journals were eligible for inclusion. Reviews, conference abstracts, non-peer-reviewed literature were excluded. Only full-length, peer-reviewed English articles were included.

### Risk of Bias Assessment

Risk of bias in the included studies was assessed using two adapted versions of the Newcastle-Ottawa Scale (NOS). Appropriate risk of bias assessment is essential for identifying systematic errors in study design, conduct, and reporting. Although previous reviews have used the modified version of the Downs and Black checklist [12, 27], there is no consensus on the best tools for assessing the risk of bias in observational studies [35–37]. The Newcastle-Ottawa Scale (NOS), however, is the most widely used due to its reliability and ease of application [38, 39]. Unlike the Downs and Black checklist, which includes items irrelevant to observational designs [40], the NOS is tailored for observational studies and evaluates three clear domains: selection, comparability, and outcome/exposure [41, 42]. Moreover, its domain-based structure aligns with contemporary systematic review guidelines that discourage composite scores [42, 43].

All studies were evaluated independently by two reviewers (FA and FS), and any disagreements between them were resolved in a consensus meeting; a third reviewer (OR) was consulted when necessary. Inter-rater agreement was evaluated using kappa statistics, with values were interpreted based on cut-off points recommended by Landis and Koch (1997): <0.00 (poor agreement); 0.00-0.20 (slight agreement); 0.21–0.40 (fair agreement); 0.41–0.60 (moderate agreement); 0.61–0.80 (substantial agreement); and 0.81-1.00 (almost perfect agreement) [44]. An adapted case-control version of the NOS was used in studies with a between-subjects design where the comparators were uninjured controls. An adapted cross-sectional version of the NOS was used for studies with a within-subjects design comparing contralateral unaffected limbs. Both versions contained three domains related to:

1) Selection of study participants (4 items in both versions of the NOS).

2) Methods to control for confounding (1 item in both versions of the NOS),

3) Methods to ascertain either exposure or outcome of interest assessment (3 items in case-control version, 2 items in cross-sectional version).

Study quality was graded using a star rating system, with case-control studies receiving a maximum of 11 stars and cross-sectional studies up to 9 stars. In studies with a between-subjects design, two groups were considered comparable if they were matched demographically (age and sex) in the study design, or if the statistical analysis was adjusted accordingly. In addition, studies that controlled for other important factors, such as pain and physical activity, received an extra star. In studies using a within-subjects design, the concept of comparability was linked to the study’s attempt to control for confounding variables, such as pain, inflammation, and laxity, when comparing the injured side to the unaffected side or the reconstructed side to the unaffected side. Case-control studies were given a score out of 11 and were categorized as follows: low quality (stars ≤ 4), moderate quality (5 ≤ stars ≤ 8), and high quality (stars ≥ 9). For studies with a within-subjects design, the maximum possible score was 9 stars and was classified as: low quality (stars ≤ 3), moderate quality (4 ≤ stars ≤ 6), and high quality (stars ≥ 7).

### Data Extraction Strategy

The following relevant information was extracted from the articles using a pre-designed data extraction form by the lead author (FA) and the accuracy of the extracted data was checked by the second reviewer (FS): Study details (first author, year of publication, design), characteristics of participants (age, sample size, sex, time since injury/reconstruction), task characteristics (contraction intensity, type of contraction, angular velocity, target level), comparators (involved limb and/or healthy controls), outcome measures, and main findings.

### Data Analysis

Means and standard deviations of force control outcomes, in addition to the sample size, were used for meta-analysis [25]. When only Standard Error (SE) was available in a study, SD was calculated using the formula SD = SE×√N. When range and median were reported, mean and SD were calculated using the approach suggested by Hozo et al. [45]. When data for different groups or graft types were reported separately in a study, the formula suggested by the Cochrane Handbook for Systematic reviews of Interventions was used to combine the baseline data of the two groups into a single group. If data were only available in figures, WebPlotDigitizer (https://automeris.io/WebPlotDigitizer) was used to extract numerical data from the figures.

All meta-analysis procedures were performed using the ‘Metan’ package in STATA MP V.14.2 (StataCorp, College Station, Texas, USA). Separate meta-analyses were conducted for each force control measure within each ACL condition (injured/reconstruction). Sub-group analyses based on comparators (unaffected limb and/or healthy controls) and isometric contraction intensity were performed when at least three studies were available. Due to the small number of studies and vast methodological heterogeneity, we were unable to conduct subgroup analyses based on time since injury/reconstruction.

Given the anticipated heterogeneity, random-effect models were used for each meta-analysis. The standardized mean difference (SMD; Glass delta) and 95% confidence intervals were used as the measure of effect size. Pooled effect sizes were interpreted according to Cohen’s guidelines: small (0.20–0.49), moderate (0.50–0.79), or large (≥ 0.80). The results of individual studies and pooled estimates of the Glass’ delta values ​​were presented using forest plots. A positive Glass’ delta value indicates impaired force control in individuals with ACL injury and/or ACLR when compared to controls or the affected limb versus the contralateral, unaffected limb. Due to the limited number of studies reporting secondary outcomes, a narrative synthesis approach was utilized.

Statistical heterogeneity among studies was assessed using the I^2^ statistic and the Cochrane Q test with significance set at *p* ≤ 0.05. The interpretation threshold of I^2^ values was as follows: 0–40% (may indicate low or unimportant heterogeneity); 30–60% (may represent moderate heterogeneity); 50–90% (may represent substantial heterogeneity); and 75–100% (considerable heterogeneity) [46]. Publication bias was examined with Egger’s linear regression test and the trim-and-fill method. The absence of a significant p-value for the intercept from the Egger regression test, along with no change in effect size using the trim-and-fill method, indicated no evidence of publication bias. We conducted a sensitivity analysis to evaluate the robustness of the pooled estimates and identify potential influential studies by sequentially excluding one study at a time and recalculating the summary effect size, along with changes in the overall effect estimate, confidence intervals, and I^2^ statistics. In addition, we excluded studies deemed poor quality to assess their potential impact on the meta-analysis results.

## Results

**Fig. 1:**
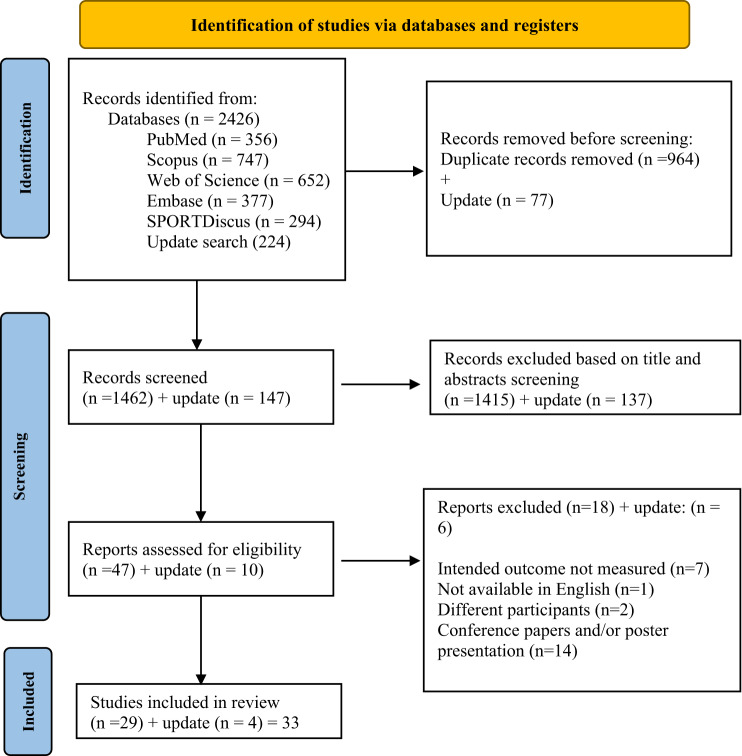
PRISMA flow diagram of the identification and selection of the studies included in this meta-analysis

The initial search yielded a total of 2426 records, as presented in the PRISMA flowchart (Fig. 1). After removing duplicates, 1462 records remained and were screened based on their titles and abstracts, resulting in the exclusion of 1415 records. The remaining 47 full-text articles were assessed, and 18 studies were excluded because they did not meet the predefined eligibility criteria [47–64]. The updated search identified 224 new records; four met the inclusion criteria after full-text review. This review included 33 studies. Figure 1 shows the exclusion criteria for those studies. Specifically, seven studies did not assess force control outcomes [47, 52, 53, 60, 64–66]. Two studies were excluded because the participants were not individuals with ACL injury and/or reconstruction [50, 67]. One study was excluded because the full text was not written in English [48]. Fourteen studies were identified as conference papers or poster presentations [49, 51, 54–59, 61–63, 68–70].

### Characteristics of Included Studies

As summarized in Table 1, of the 33 studies, 20 recruited individuals with ACLR [21, 22, 25, 28–30, 32–34, 71–81], 12 included individuals with ACL injury [26, 31, 82–91], and one study included both ACLR and ACL injury [92]. Seven studies used a cross-sectional design [28, 31, 80, 84, 87–89], 17 used a case-control design [21, 22, 25, 26, 30, 32, 33, 71–74, 77, 81, 82, 85, 86, 92], one was a randomized controlled trial [83], five conducted a prospective longitudinal design [29, 34, 78, 90, 91], and three had a pretest-posttest design [75, 76, 79]. Twelve studies included comparative data from uninjured control groups [21, 22, 26, 32, 73–79, 85], 12 studies from the contralateral unaffected limbs [28, 29, 31, 34, 80, 83, 84, 87–91], and nine studies from both contralateral unaffected limbs and uninjured controls [25, 30, 33, 71, 72, 81, 82, 86, 92].

**Table 1:** Characteristics of the included studies

Author; year of publication	Study design	ACL status/ graft type	Participants characteristics	Comparison group	Time elapsed since injury/reconstruction	Force control task; type of contraction; contraction intensity; test position, type of apparatus; recording time	Force control measure	results
San Martín-Mohr et al. [73]	Case-control	ACL Reconstruction; BPTB; HT	56 individuals with ACL reconstruction; sex: men; Age: 26.15 ± 5.06 years; height: 174 ± 3 cm; Weight: 75.43 ± 8.7 kg; 27 healthy controls; sex: men; age: 24.27 ± 3.28 years; height: 176 ± 6 cm; Weight: 75.9 ± 8.53 kg	Healthy subjects	8.3 ± 2.34 months	Isometric contraction using load cell anchored to the distal end of the limb in sitting position with 90° of knee flexion; 15% MVIC; constant target; 10-second	CoV (%)	No significant differences between ACLR group and healthy control group
Ward et al. [26]	Case-control	ACL injury	18 individuals with ACL injury; sex: 6 women, 12 men; age: 29.6 ± 8.4; height: 174 cm ± 7 cm, weight: 76 ± 10.4 kg; 18 healthy controls; sex: 6 women, 12 men; age: 29.2 ± 6.8 years; height: 179 ± 7 cm; weight: 79 ± 8.4 kg	Healthy subjects; and contralateral uninvolved limb	2.31 ± 1.41 months	Isometric; sinusoidal target with the contraction intensity of 5% to 25% of body weight using a force transducer in sitting position with 60° of knee flexion and 90 ° of hip flexion; 60-second	RMSE	Higher RMSE in both the involved and uninvolved limbs of ACL injury individuals compared to healthy control group
Hollman et al. [86]	Case-control	ACL injury	24 individuals with ACL injury; sex: 12 women; 12 men; age: 18.8 ± 3.1 years; height: 173.6 ± 9.4 cm; weight: 75.8 ± 14.3 kg; 25 healthy controls; sex: 14 women, 11 men; age: 18.8 ± 3.1 years; height: 173.9 ± 8.5 cm; weight: 75.8 ± 14.3 kg	Healthy subjects; and contralateral uninvolved limb	1.83 ± 2.20 months	Isometric contractions, constant target 10%, 25%,35%,50% MVIC using MLP-300 load cell while sitting on a dynamometry chair with 90° of knee flexion, 10-second	CoV (%); Fractal scaling exponents	No significant differences in CoV measures between ACL deficient group and healthy group, No significant difference between involved and un-involved limbs. Higher the fractal scaling exponent in the injured than non-injured limb at the 25% and 35% effort levels; higher the fractal scaling exponent at the 35% effort level in ACL deficient group than in the control group
Telianidis et al. [74]	Case-control	ACL reconstruction; STGT graft	28 ACL reconstruction; sex: 19 men, 9 women; Age: 27 ± 5 years; height: 177 ± 11 cm; weight: 78 ± 16 kg; 29 healthy controls; sex: 15 men, 14 women; age: 23.8 ± 4.1 years; height: 171.5 ± 7.4 cm; weight: 69.3 ± 10.6 kg	Healthy subjects	17 ± 2 months	Isometric contraction, sinusoidal target between 5% and 30% MVIC using isokinetic kin-com dynamometer while sitting with 90 ° hip flexion and 60° knee flexion; one minute	RMSE	Higher RMSE in ACL reconstruction group compared to the control group
Spencer et al. [80]	Cross-sectional	ACL reconstruction; BPTB	28 ACL reconstruction; sex: 14 women, 14 men; age: 20 ± 5 years; height: 177.8 ± 9.8 cm; weight: 72.2 ± 10.6 kg	Contralateral uninvolved limb	6 months	Isometric contraction, constant target at maximal effort using Biodex system IV isokinetic dynamometer while sitting at 90° of hip flexion and 90° of knee flexion; 5-second	CoV (%); SD	Higher CoV in the ACL reconstructed limb compared to the contralateral limb; lower SD in the ACL reconstructed limb compared to the contralateral limb
Perraton et al. [21]	Case-control	ACL reconstruction; four-strand hamstring and gracilis tendon autograft	66 ACL reconstruction; sex: 23 women, 43 men; age: 28.4 ± 6.2 years; height: 175 ± 1 cm; weight: 78.1 ± 14.7 kg; 41 healthy controls; sex: 16 women, 25 men; age: 25.8 ± 5.3 years; height: 174 ± 1 cm; weight: 72.5 ± 11.1 kg	Healthy subjects	18 ± 3 months	Isometric contraction; sinusoidal target between 5% to 30% MVIC using Kin-Com isokinetic dynamometer while sitting position with 90 ° of hip flexion and 60° of knee flexion; one minute	RMSE	Higher RMSE in the group with ACL reconstruction compared to the control group
Chaney et al. [28]	Cross-sectional	ACL reconstruction	102 ACL reconstructed individuals; sex: 44 men, 58 women, age: 21.6 ± 13.7 years; height: 169.9 ± 13.9 cm, weight: -	Contralateral uninvolved limb	10 ± 1 months	Isokinetic contractions at maximal effort and 60°/sec. using Humann NORN isokinetic dynamometer	Determinism (DET); entropy (ENTR)	Lower DET and ENTR in the involved limb than the uninvolved limb
Goetschius and Hart [22].	Case-control	ACL reconstruction	53 individuals with ACL reconstruction; sex: 27 men, 26 women; age: 23.4 ± 4.9 years; height: 170 ± 1 cm; weight: 74.6 ± 14.8 kg; 50 healthy controls; sex: 28 men, 22 women; age: 23.3 ± 4.4 years; height: 170 ± 1 cm; weight: 67.4 ± 13.2 kg	Healthy subjects	44.1 ± 29.9 months	Isometric contraction with constant target at maximal effort using Biodex system III dynamometer while sitting using Biodex system with 85° of hip flexion and 90° flexion of knee; one minute	CoV (%)	Higher CoV in the ACL reconstructed group compared to the control group
Bodkin et al. [25]	Case-control	ACL reconstruction; patellar tendon graft (*n* = 76), hamstring graft (*n* = 44)	120 individuals with ACL reconstruction; sex: 65 women, 55 men; age: 21 ± 8.2 years, height: 171.8 ± 11 cm; weight: 73.8 ± 17.5 kg; 95 healthy controls; sex: 50 women, 45 men; age: 21.5 ± 2.9 years; height: 174.1 ± 11 cm; weight: 70.7 ± 11.4 kg	Healthy subjects; contralateral uninvolved limb	5.96 ± 0.48 months	Isometric contraction at maximal effort with constant target using Biodex system IV dynamometer while sitting with 80° flexion of trunk and 90° flexion of knee; 30-second	Approximate entropy (ApEn)	Greater ApEn in the reconstructed limb compared to the contralateral uninvolved limb and also compared to the healthy control group
Johnson et al. [91]	Longitudinal prospective cohort	ACL injury (the baseline data, pre-surgery, were used)	34 individuals with ACL injury; sex: 19 women, 15 men; age: 16.5 ± 2.7 years; height: 172 ± 10 cm; weight: 75.5 ± 18.6 kg;	Contralateral uninvolved limb	2.52 ± 1.75 months	Isometric contraction at maximal effort with constant target using Biodex system while sitting with 90 ° flexion of hips and knees; 5-secnd	Sample entropy	Quadriceps sample entropy was not significantly different between involved and uninvolved limbs at baseline (pre-surgery)
Niederer et al. [76]	Pretest-posttest	ACL reconstruction; hamstring tendon autograft	19 individuals with ACL reconstruction; sex: 10 women, 9 men; age: 25.7 ± 4.2 years; height: 176 ± 9.5 cm; BMI: 23.3 ± 2.8 kg/m^2^; 19 healthy controls; sex: 10 women, 9 men; age: 24.8 ± 1.6 years; height: 175 ± 8.7 cm; BMI: 22.9 ± 1.8 kg/m^2^	Healthy controls	38 ± 19 month	Isometric contraction at maximal effort with constant target using m^3^ (multi muscle machine) while sitting; the middle (out of 10 s) 5-second	Absolute variability; coefficient of variation (CoV)	There were no significant differences between the ACLR group and the healthy control group
Bryant et al. [92]	Case-control	ACL injury, and ACL reconstruction; bone-patellar tendon-bone graft	13 individuals with ACL injury; sex: 3 women, 10 men; age: 32.7 ± 8.6 years; height: 174.5 ± 6.2 cm; weight: 25 individuals with ACL reconstruction; sex: 11 women, 14 men; age: 30.5 ± 8.1 years; height: 172.7 ± 10.1 cm; weight: 74.3 ± 21.5; 33 healthy controls; sex: 11 women, 22 men; age: 29.5 ± 8.8 years; height: 174.3 ± 8.3 cm; weight: 73.3 ± 14.6 kg	Both healthy control and contralateral limb	ACL injury: 75.6 ± 72.5 months; ACLR: 15.7 ± 5.5 months	Isokinetic contraction at maximal effort at 180 °/sec., using Cybex dynamometer while sitting	The wavelet-derived mean instantaneous frequency of the extension-torque time curve	Higher the mean instantaneous frequency of the extension torque for the subjects with ACL injury and the subjects with a reconstructed ACL; higher the mean instantaneous frequency of the extension torque for the involved limb of the subjects with ACL injury or ACL reconstruction in comparison with their uninvolved limb
Goetschius et al. [75]	Pretest-posttest (only baseline data were used)	ACL reconstruction	32 ACL reconstruction; sex: 14 women, 18 men; age: 24.1 ± 4.9 years; height: 171.9 ± 12.2 cm; weight: 73.3 ± 14.8 kg; 32 healthy controls, age: 24.3 ± 4 years; height: 172.2 ± 10.2 cm; weight; 70.4 ± 13.6 kg	Healthy controls	45.1 ± 37.4 months	Isometric contraction at maximal effort, constant target using Biodex system III dynamometer while sitting with 85° hip flexion and 90° knee flexion,3-second	CoV (%)	Greater CoV in ACLR groups compared with healthy controls
Pua et al. [88]	Cross-sectional	ACL injury	87 individuals with ACL injury; sex: 73 men, 14 women; age: 26 ± 5.8 years; BMI: 23.8 ± 3.3 kg	Contralateral uninvolved limb	1.86 months (range: 0.7 to 5.6)	Isokinetic contraction at maximal effort at 60°/s using Biodex system IV dynamometer while sitting with 90° of hip flexion	Wavelet-derived mean instantaneous frequency	Higher torque frequency level in the ACL injured limb compared with the contralateral limb
Skurvydas et al. [87]	Cross-sectional	ACL injury	13 individuals with ACL injury; sex: men; age: 30.1 ± 9.7 years; height: 183.9 ± 8.8 cm; weight: 94.4 ± 11.8 kg;	Contralateral uninvolved limb	1.2 ± 0.55 months	Isometric contraction at 20% of MVIC with constant target using Biodex system III dynamometer while sitting; during 20 s contraction	CoV (%), and permutation entropies (PE)	No significant differences between the ACL injured limb and the uninvolved limb in terms of CoV; lower permutation entropies in the involved limb than the uninvolved limb
Tsepis et al. [89]	Cross-sectional	ACL injury	30 individuals with ACL injury; sex: men; age: 27.7 ± 7.3 years; height: 176 ± 7.9 cm; weight: 78.6 ± 9.7 kg	Contralateral uninvolved limb	32 months (range: 2 to 180 months)	Isokinetic contraction at maximal effort at 60°/s using Biodex system III dynamometer while sitting with 85° of hip flexion and 90 ° knee flexion	Frequency content using fast Fourier transform analysis	Higher frequency content in the injured limb compared with the uninvolved limb
Baumeisteret al. [77]	Case-control	ACL reconstruction; quadrupled hamstring tendon autograft	9 individuals with ACL reconstruction; sex: 2 women, 7 men; age: 25 ± 5 years; height: 182 ± 10 cm; weight: 76.8 ± 12.2 kg; 9 healthy controls; sex: 2 women, 7 men; age: 24 ± 3 years; height: 181 ± 9 cm; weight: 73 ± 10.2 kg	Healthy control	12 ± 4.7 months	Isometric contraction at 50% of MVIC, constant target, using M3 training machine; while sitting with 110° of the hip flexion and 90° of the knee flexion	Aberration (error) during the force reproduction task	No significant differences between the ACLR group and the healthy control group
Cobian et al. [29]	Prospective longitudinal study	ACL reconstruction; 27 individuals with bone-patellar tendon-bone autografts, 1 individual with quadriceps tendon autograft, 1 individual with hamstring tendon autograft, 1 individual with Achilles tendon allograft	30 individuals with ACL reconstruction; sex: 16 women, 14 men; age: 20.1 ± 1.4 years; height: 176.3 ± 10.7 cm; weight: 79.7 ± 21.7 kg	Contralateral uninvolved limb	4.1 ± 0.6 months	Isometric contraction at maximal effort with constant target using Biodex system IV dynamometer while sitting with 85° of the hip flexion and 90° of the knee flexion; 3–4 s	The mean difference between initial and low-pass filtered torque signals was expressed as a percentage of peak torque	Significant differences between involved and uninvolved limbs at 4 and 6 months, there was no significant differences between involved and uninvolved limb at 12 months
Zult et al. [82]	Case-control	ACL injury	32 individuals with ACL injury; sex: 16 women, 16 men; age: 23 ± 4 years; height: 178 ± 9 cm; weight: 77 ± 12 kg; 20 healthy controls; sex: 10 women, 10 men; age: 22 ± 2 years; height: 178 ± 11 cm; weight: 73 ± 12 kg	Both healthy control, and contralateral uninvolved limb	6.93 ± 4.83 months	Isometric contraction at 20% of MVIC with constant target, and isokinetic contraction at 60°/s between 90° and 10° of the knee flexion using custom build dynamometer while sitting with the hips and knees in 90° flexion; 5-second recording time	Absolute error, and CoV	No significant differences between the two groups and or between the injured limb and the uninvolved limb of the individuals with ACL injury
Zult et al. [83]	Randomized controlled trial	ACL injury	43 individuals with ACL injury; sex: 20 women, 24 men; age: 28 ± 9.38 years; height: 178.58 ± 7.85 cm; weight: 78.09 ± 12.18 kg	Contralateral uninvolved limb	5.82 ± 3.94 months	Isometric contraction at 20% of MVIC with constant target while sitting with 65° of the knee flexion; isokinetic contraction at 60°/s between 10° and 90° of knee flexion, using Biodex dynamometer, 5-second duration of recording time	Absolute error, CoV	No significant differences in force steadiness between the injured limb and the uninvolved limb at the baseline (pre-treatment session)
Nuccio et al. [30]	Case-control	ACL reconstruction; BPTB (*n* = 8), and STGR (*n* = 3)	11 individuals with ACLR; men; age: 24.8 ± 3.2 years; BMI: 23.3 ± 0.6 kg/m^2^; 9 healthy controls; male; age: 25.7 ± 2.5 years; BMI: 22.9 ± 0.5 kh/m^2^	Both healthy controls and contralateral limb	8.33 ± 2.73	Isometric contraction at 10% and 30% of MVIC with constant target using KinCom dynamometer while sitting, 60-second duration of recording time	CoV (%)	No significant differences in force steadiness between the ACLR group and the healthy control group, no significant differences in force steadiness between the ACL reconstructed limb and the contralateral uninvolved limb
Nemati et al. [84]	Cross-sectional	ACL injury	13 individuals with ACL injury; sex: men; age: 27.85 ± 7.04; BMI: 23.63 ± 2.25 kg/m^2^	Contralateral uninvolved limb	1.60 ± 0.71 months	Isometric contraction at 30%, and 50% of MVIC with constant target, using Biodex system IV dynamometer while sitting, 7-second duration of recording time	SD, RMSE	No significant differences in SD, and or RMSE between the injured limb and the uninvolved limb
Czaplicki et al. [78]	Longitudinal study	ACL reconstruction; four-strand Semitendinosus and Gracilis tendon graft	22 individuals with ACL reconstruction; sex: men; age: 26.5 ± 3.2 years; height: 178.6 ± 5.2 cm; weight: 79.1 ± 8.9 kg; 20 healthy controls; sex: men; age: 22.3 ± 0.9 years; height: 179.3 ± 5.2 cm; weight: 74.7 ± 7.1 kg	Healthy control group	3 time-points: 3 ,6, and 12 months after surgery	Isokinetic contraction at maximal effort and 60°/s using the Biodex system III dynamometer while sitting	The orthogonal Daubechies 4 (Db 4) and biorthogonal Bior 3.1 Wavelets	Significant differences in terms of Db 4 wavelet, and the Bior 3.1 wavelet between the healthy control group and the ACLR patients (higher values for ACLR group) only at 6th month after reconstruction
Scheurer et al. [81]	Case-control	ACL reconstruction; PT (*n* = 4), HT (*n* = 9), allograft (*n* = 2), repair (*n* = 1)	16 individuals with ACL reconstruction; sex: 8 women, 8 men; age: 20.4 ± 1.8 years; height: 174.5 ± 9.1 cm; weight: 78.7 ± 19.2 kg; 16 healthy controls; 8 women, 8 men; age: 21 ± 1.7 years; height: 174.5 ± 7.9 cm; weight: 75.9 ± 9.3 kg	Both healthy controls and contralateral uninvolved limb	33.9 ± 26.1 months	Isometric contraction at maximal effort (100% MVIC) with constant target, using Biodex system IV dynamometer while sitting with 90° of the knee flexion	CoV	No difference between the two sides nor the two groups
Rice et al. [79]	Pretest-posttest design	ACL reconstruction; Semitendinosus graft	14 individuals with ACL reconstruction; sex: 8 women, 6 men; age: 24 ± 7 years; height: 171 ± 9 cm; BMI: 24 ± 2.8 kg/m^2^; 15 healthy subjects; 8 women, 7 men; age: 25 ± 7 years; height: 174 ± 9 cm; BMI: 22 ± 2.5 kg/m^2^	Healthy controls	10 ± 5 months	Force matching task: isometric contraction with constant target at 25% and 50% of MVIC, 15-second duration recording time; force modulation task: isometric contraction with sinusoidal force modulation task between 20% − 80% of the MVIC; 60-second duration of recording time	RMSE	Greater RMSE for the ACLR group compared to the healthy controls
Scoz et al. [90]	Prospective diagnostic study	ACL injury	29 individuals with ACL injury; 13 women, 16 men; age: 36.29 ± 12.49 years; height: 172 ± 8 cm; weight: 74.87 ± 14.41 kg	Contralateral limb	3.12 ± 1.12 months	Concentric contraction at maximal effort and 60°/s angular velocity for a 100° arc of motion using Humac-Norm dynamometer while sitting with 85° of the hip flexion	Frequency content using fast Fourier transform analysis	Higher oscillations and more unstable force output of the injured knees
Sherman et al. [72]	Case-control	ACL reconstruction: BTB (*n* = 10), HT (*n* = 5), QT (*n* = 4), allograft (*n* = 1)	20 individuals with ACLR; 12 women, 8 men; age: 21.2 ± 2.2 years; height: 174.4 ± 9.7 cm; weight: 78.3 ± 16 kg; 20 healthy subjects; 12 women, 8 men; age: 21.5 ± 2.2 years; height: 173.4 ± 11.1 cm; weight: 75 ± 16.7 kg	Both healthy controls and contralateral uninvolved limb	15.1 ± 9.6 months	Isometric contraction at 50% of the MVIC with constant target using Biodex system IV dynamometer while sitting with the 85° of the hip flexion and 90° of the knee flexion, 15-second duration of recording time	RMSE; CoV	Higher RMSE in the ACLR group compared to the control group. No significant differences between the two groups in terms of CoV, no significant differences in CoV and or RMSE between the reconstructed limbs and the uninvolved limb
Sherman et al. [71]	Case-control	ACL reconstruction: BTB (*n* = 7), HT (*n* = 7)	14 individuals with ACLR; 7 women, 7men; age: 20.9 ± 2.5 years’ height: 177.8 ± 9.2 cm; weight: 73.4 kg; 13 healthy subjects; 6 women, 7 men, age: 21.8 ± 1.8 years; height: 173.2 ± 11.4 cm; weight: 74.1 ± 13.1 kg	Both healthy controls and contralateral uninvolved limb	28.7 ± 24.5 months	Isometric contractions at 30%, 50%, 70%, and 100% MVIC with constant target using Biodex system 4 Pro dynamometer while sitting with the 85° of the hip flexion and 90° of the knee flexion, 15 s for 30%, and 50% MVIC; 10 s for 70% MVIC; 5 s for 100% MVIC	Normalized RMSE	During contractions at 100% effort, the ACLR group demonstrated higher nRMSE than the controls
Amirshakeri et al. [85]	Case-control	ACL injury	26 individuals with ACL injury, sex: men; age: 25.2 ± 1.7 years; height: 176.57 ± 7.32 cm; weight: 77.28 ± 8.82 kg; 26 healthy subjects; sex: men; age: 25.3 ± 2.52 years; height: 175.49 ± 7.64 cm; weight: 77.47 ± 9.43 kg	Healthy controls	Inclusion criteria: 4 to 6 months since injury	Isometric contraction at 50% of the MVIC with constant target; using load cells while sitting with 110° of the thigh flexion and 60° of the knee flexion, 8-second duration of recording time	Absolute error	No significant difference in the force perception error between the control group and the patients with ACL injury when target force was reproduced on remembered ipsilateral side; higher force perception errors in the patients with ACL injury when target force was produced on contralateral side. either remembered or concurrently
Hunt et al. [32]	Case-control	ACL reconstruction; graft type ?	12 individuals with ACL reconstruction; sex ?; age: 23.25 ± 4 years; height: 171 ± 9 cm; weight: 78.75 ± 3.20 kg; 13 healthy subjects; sex ?; age: 22.38 ± 1.94; height: 175 ± 10 cm; weight: 73.68 ± 11.3 kg	Healthy controls	?	Isometric contraction at maximal level with constant target using isokinetic dynamometer machine (Human Norm) while sitting with 90° of the hip flexion and 60° of the knee flexion; 5 s duration of recording time	CoV; Frequency domain related parameters: Peak power frequency; total power; mean frequency, median frequency	No significant differences between two groups in terms of CoV, mean and median of frequency; Greater peak power frequency (less steadiness) in ACL reconstruction group than control group
Lemos et al. [31]	Cross-sectional	ACL injury	42 individuals with ACL injury; sex: 14 women, 28 men; age: 23 years (20–27 years); height: 173 cm (169–177 cm); weight: 70 kg (64–78 kg);	Contralateral uninvolved limb	?	Isometric contraction at maximal level with constant target using isokinetic dynamometer machine (Human Norm) while sitting with 85° of the hip flexion; 3–5 s duration of recording time (a region-of-interest of 1.5 s in the middle of the torque curve was defined	CoV; sample entropy	No significant difference in CoV between injured limb and contralateral uninvolved limb; higher sample entropy of force signal produced by the injured limb than contralateral uninvolved limb; a moderate negative correlation between single hop test and sample entropy of force signal
Sherman et al. [33]	Case-control	ACL reconstruction; BPB (*n* = 10), HT (*n* = 5), QT (*n* = 4), allograft (*n* = 1)	20 individuals with ACL reconstruction; sex: 12 women, 8 men; age: 21.2 ± 2.2 years; height: 174.4 ± 9.7 cm; weight: 78.3 ± 16 kg; 20 healthy individuals; sex: 12 women, 8 men; age: 21.5 ± 2.2 years; height: 173.4 ± 11.1 cm; weight: 75 ± 16.7 kg	Healthy controls, and contralateral uninvolved limb	15.1 ± 9.6 months	Isometric contraction at 50% of the MVIC with constant target using Biodex system 4 Pro isokinetic dynamometer while sitting with 85° of hip flexion, and 90 ° of the knee flexion; 15 s duration of recording time	CoV; normalized RMSE	No significant differences between two groups, or between involved and non-involved limbs
Jo and Kim [34]	prospective interventional study (only baseline data were used)	ACL reconstruction; graft type ?	10 individuals with ACL reconstruction; sex: men; age: 24.1 ± 1.36 years; height: 176.83 ± 1.34 cm; weight: 78.03 ± 3.69 kg	Contralateral uninvolved limb	10.60 ± 2.16 months	Isometric contraction at 70% of the MVIC with constant target using a load cell with a built-in strange gauge while sitting at 90° of the knee flexion; 15 s duration of recording time (the middle 8 s of the signal were analyzed)	CoV	Higher CoV of force produced by involved limb than contralateral uninvolved limb

ACL: Anterior Cruciate Ligament, ACLR: Anterior Cruciate Ligament Reconstruction, SD: Standard Deviation, CoV: Coefficient of Variation, RMSE: root Mean Square Error, DET: Determinism, ENTR: Entropy, PE: Permutation Entropy, APEN: Approximate Entropy, Db4: Daubechies, Bior: Biorthogonal, BPTB: Bone Patellar Tendon Bone, HT: Hamstring tendon, STGT: Semitendinosus Gracilis Tendon

The total sample consisted of 1661 participants: 550 healthy individuals, 707 with ACLR, and 404 with ACL injuries. One study did not specify participants’ sex [32]. Eight studies included only male participants [30, 34, 73, 78, 84, 85, 87, 89], while the remaining studies included both sexes. The average age of participants ranged from 16.5 to 36.3 years. The average time since injury varied from 1.2 to 75.6 months, and the average time since reconstruction ranged from 3 to 75.6 months. Five studies did not report graft type [22, 28, 32, 34, 75]. Two studies included individuals who received autogenous bone-patellar tendon-bone (BPTB) [80, 92], while six studies recruited individuals with a hamstring tendon (HT) graft [21, 65, 74, 76–79]. Three studies included a mix of individuals; some received BPTB or HT [30, 71, 73]. One study recruited individuals who had received a patellar tendon graft or HT graft [25]. Four studies included individuals with BPTB, HT, quadriceps tendon, and allografts [29, 33, 72, 81].

Regarding the force control task, 25 studies assessed force control during isometric contractions, 22 studies utilized a constant target [22, 25, 29–34, 71–73, 75–77, 79–81, 84–87, 91] and three studies with a sinusoidal target [21, 26, 74]. Six studies assessed force control during isokinetic contractions [28, 78, 88–90, 92], and two studies employed both isometric and isokinetic dynamometry [82, 83]. Coefficient of variation (CoV) and root mean square error (RMSE) emerged as the preferred metrics for assessing force control in most studies. Six studies reported frequency content [32, 78, 88–90, 92], while some studies assessed the complexity of the muscle force signal using a nonlinear dynamics approach [25, 28, 31, 86, 87, 91]. Five studies also investigated the association between force control measures and functional outcomes [21, 22, 31, 88, 92].

### Risk of Bias Assessment

#### Studies with a Between-Subjects Design

The initial risk of bias assessment before consensus showed almost perfect inter-rater agreement (kappa = 0.82). Among studies with a between-subjects design, scores ranged from 4 to 8 out of 11 stars. Two studies were rated as “low” quality [73, 86], while the remaining 19 (out of 21 were rated as “moderate” quality (Table 2). Out of 21 studies, 20 clearly defined control groups, and 18 met the criteria for case definition [21, 22, 25, 26, 33, 71–79, 81, 82, 85, 92]. Only two studies reported non-response rates [71, 74], and only one study met the criteria for representativeness of the cases within the sampled population [26].

**Table 2:** Risk of bias assessment based on the adapted case-control version of the Newcastle - Ottawa Scale for between subjects’ comparison Studies

Study	Selection (max 4 stars)				Comparability (max 2 stars)		Exposure/ Outcome measurement (max 5 stars)			Overall quality
	Case definition	Representativeness of the cases	Selection of controls	Definition of controls	Age/ gender	Physical activity / pain	Ascertainment of exposure^a^	Assessment of the outcome^b^	Non-response rate	
Martin-Mohr et al. [73]	✮		✮	✮			✮✮	✮		Low
Ward et al. [26]	✮	✮	✮	✮			✮	✮		Moderate
Hollman et al. [86]			✮	✮		✮	✮✮	✮		Low
Telianidis et al. [74]	✮			✮			✮✮	✮✮	✮	Moderate
Perraton et al. [21]	✮		✮	✮			✮	✮✮		Moderate
Goetschius and Hart [22]	✮		✮	✮	✮		✮	✮✮		Moderate
Bodkin et al. [25]	✮		✮	✮			✮	✮✮		Moderate
Niederer et al. [76]	✮			✮	✮	✮	✮✮	✮✮		Moderate
Bryant et al. [92]	✮			✮	✮	✮	✮	✮✮		Moderate
Goetschius et al. [75]	✮		✮	✮			✮	✮		Moderate
Baumeister et al. [77]	✮			✮	✮	✮	✮✮	✮✮		Moderate
Zult et al. [82]	✮			✮	✮	✮	✮	✮✮		Moderate
Nuccio et al. [30]			✮	✮		✮	✮	✮		Moderate
Czaplicki et al. [78]	✮			✮			✮	✮✮		Moderate
Rice et al. [79]	✮			✮	✮		✮	✮✮	✮	Moderate
Sherman et al. [72]	✮			✮	✮	✮	✮	✮✮		Moderate
Sherman et al. [71]	✮			✮	✮	✮	✮	✮✮		Moderate
Scheurer et al. [81]	✮			✮	✮		✮	✮✮		Moderate
Amirshakeri et al. [85]	✮			✮	✮	✮	✮✮	✮		Moderate
Hunt et al. [32]				✮	✮		✮	✮✮		Moderate
Sherman et al. [33]	✮		✮	✮	✮	✮	✮	✮✮		Moderate

^A^Ascertainment of exposure via structured injury data: awarded 2 stars; by structured interview: awarded 1 star

^B^Assessment of the outcome via independent blind assessment using objective validated laboratory methods, or by unblinded assessment using objective validated laboratory methods: awarded 2 stars; used non-standard or non-validated laboratory methods with gold standard: awarded 1 star

#### Studies with a Within-Subjects Design

In studies with a within-subject design, scores ranged from 4 to 8 out of 9 stars (Table 3). Three of the 12 studies were rated as “high” quality [83, 88, 90], while the other nine were rated as “moderate” quality [28, 29, 31, 34, 80, 84, 87, 89, 91]. All studies met the criteria for appropriate statistical methods and outcome assessment. In three studies [28, 83, 88], the samples were representative of the average target population. Only one study met the criteria for controlling for confounders [90], while another study addressed the response rate and comparability between respondents and non-respondents [88].

**Table 3:** Risk of bias based on the adapted cross-sectional version of the Newcastle-Ottawa Scale for within-subjects comparison

Study	Selection (max 5 stars)				Comparability (max 1 star)	Outcome (max 3 stars)		Overall quality
	Representativeness of the sample	Sample size	Non-respondents	Ascertainment of the exposure ^a^	Confounding (symptoms: pain/laxity)	Assessment of the outcome ^b^	Statistical test	
Spencer et al. [80]				✮✮		✮✮	✮	Moderate
Chaney et al. [28]	✮			✮✮		✮✮	✮	Moderate
Johnson et al. [91]		✮		✮		✮✮	✮	Moderate
Pua et al. [88]	✮	✮	✮	✮✮		✮✮	✮	High
Skurvydas et al. [87]		✮		✮		✮✮	✮	Moderate
Tsepis et al. [89]				✮✮		✮✮	✮	Moderate
Cobian et al. [29]				✮✮		✮✮	✮	Moderate
Zult et al. [83]	✮	✮		✮✮		✮✮	✮	High
Nemati et al. [84]				✮✮		✮✮	✮	Moderate
Scoz et al. [90]		✮		✮✮	✮	✮✮	✮	High
Lemos et al. [31]		✮		✮✮		✮✮	✮	Moderate
Jo and Kim [34]		✮		✮		✮✮	✮	Moderate

^A^ Ascertainment of the exposure via structured injury data: awarded 2 stars, via structured interview: awarded 1 star

^B^ Assessment of the outcome by independent blind assessment using objective validated laboratory methods or via unblinded assessment using objective validated laboratory methods: awarded 2 stars; using non-standard or non-validated laboratory methods with gold standard: awarded 1 star

### Publication Bias Assessment

Egger’s regression test for the meta-analysis of force control assessed by CoV in the subgroup comparing individuals with ACL injury to uninjured controls (*p* = 0.014, Intercept = 18.13, 95% CI = 6.86 to 29.4), for the meta-analysis of force control assessed by RMSE in subgroup comparing reconstructed knee with contralateral unaffected knee (*p* = 0.003, Intercept = 13.13, 95% CI = 7.49 to 18.77), and for the meta-analysis of force control assessed by frequency content in individuals with ACLR compared to uninjured controls (*p* = 0.001, Intercept = 5.95, 95% CI = 3.13 to 8.78) detected evidence of publication bias. However, Egger’s regression results should be interpreted with caution in analyses with fewer than ten comparisons.

The trim-and-fill method suggested missing two studies from the meta-analysis of force control assessed by RMSE in individuals with ACL injury compared to uninjured controls. The adjusted effect size was null (SMD = − 0.083, 95% CI= -1.02 to 0.85); the initial meta-analysis also yielded a non-significant pooled effect (SMD = 0.653, 95% CI= -0.22 to 1.52). However, the extreme shift in the point estimate from positive to null reveals that the original positive trend was an artifact of bias. Furthermore, the trim-and-fill method indicated that two studies were missing from the meta-analysis of force control assessed by RMSE in ACLR compared to the contralateral unaffected knee; the adjusted effect size was non-significant (SMD= -0.225, 95% CI= -0.60 to 0.15), likewise the initial meta-analysis found a negligible, non-significant effect (SMD= -0.02, 95% CI= -0.37 to 0.33). The available evidence, even after adjusting for publication bias, is consistent with a null effect.

In the meta-analysis of assessing frequency content in individuals with ACLR compared to uninjured controls, three studies were found to be missing. The adjusted effect size was significantly large (SMD = 1.062, 95% CI = 0.40 to 1.71); however, its magnitude is substantially more conservative, the initial meta-analysis indicated a very large effect (SMD = 1.57, 95% CI = 0.98 to 2.16). This indicates that previous studies likely overestimate the true effect size due to the absence of smaller studies. No studies were imputed for other analyses, and the pooled Glass delta values ​​did not change, suggesting that the potential risk of publication bias was not considerable.

### Force Control in ACL Injury

Thirteen studies assessed quadriceps force control in individuals with ACL injury, comparing the affected limbs to the unaffected contralateral limb (*n* = 8) [31, 83, 84, 87–91], uninjured controls (*n* = 1) [85], or both the unaffected contralateral limb and uninjured controls (*n* = 4) [26, 82, 86, 92]. Except for one study [26], the remaining studies that assessed quadriceps force control using one of the measures of SD, CoV, RMSE, and AE found no significant differences between the involved limb of individuals with ACL injury and their uninvolved contralateral limb or the uninjured controls.

Only one study [26] assessed quadriceps force control ability using a sinusoidal target, reporting higher RMSE in both affected and unaffected limbs of individuals with ACL injury compared to uninjured controls. Also, in the study by Amirshakeri et al. [85], the force perception error was greater among individuals with ACL injury than in the uninjured control group. However, this was only observed when the target force was generated on the contralateral side. Zult et al. [82, 83] assessed force control during both isometric and isokinetic contractions. However, we included only isometric measurements in the statistical analysis to minimize heterogeneity for the meta-analysis. In addition, studies evaluating the complexity of the force signal were excluded from the meta-analysis due to variability in outcome measures.

Findings on signal complexity were mixed. Johnson et al. [91] found that the sample entropy of the maximum torque produced by the quadriceps did not differ between the affected and unaffected contralateral limbs. In contrast, Lemos et al. [31] reported higher sample entropy of maximum torque produced by the affected limb compared to the unaffected contralateral limb. Additionally, Skurvydas et al. [87] found that the permutation entropy of quadriceps torque at 20% MVIC was lower in the affected limb compared to the unaffected contralateral limb. Hollman et al. [86] also reported that the fractal scaling exponent at 25% and 35% MVIC was higher in the affected limb than in the unaffected contralateral limb. In addition, at 35% MVIC, the fractal scaling exponent in the affected limb was higher than in the uninjured control group.

Four studies [88–90, 92] examined the frequency contents of quadriceps torque time curves using fast Fourier and/or wavelet transformation during isokinetic contractions at angular velocities of 60°/s (*n* = 3) [88–90] and 180°/s (*n* = 1) [92]. All four studies reported higher torque frequency levels in the affected limb compared with the unaffected contralateral limb.

### Results of Meta-Analyses on ACL Injury

**Fig. 2:**
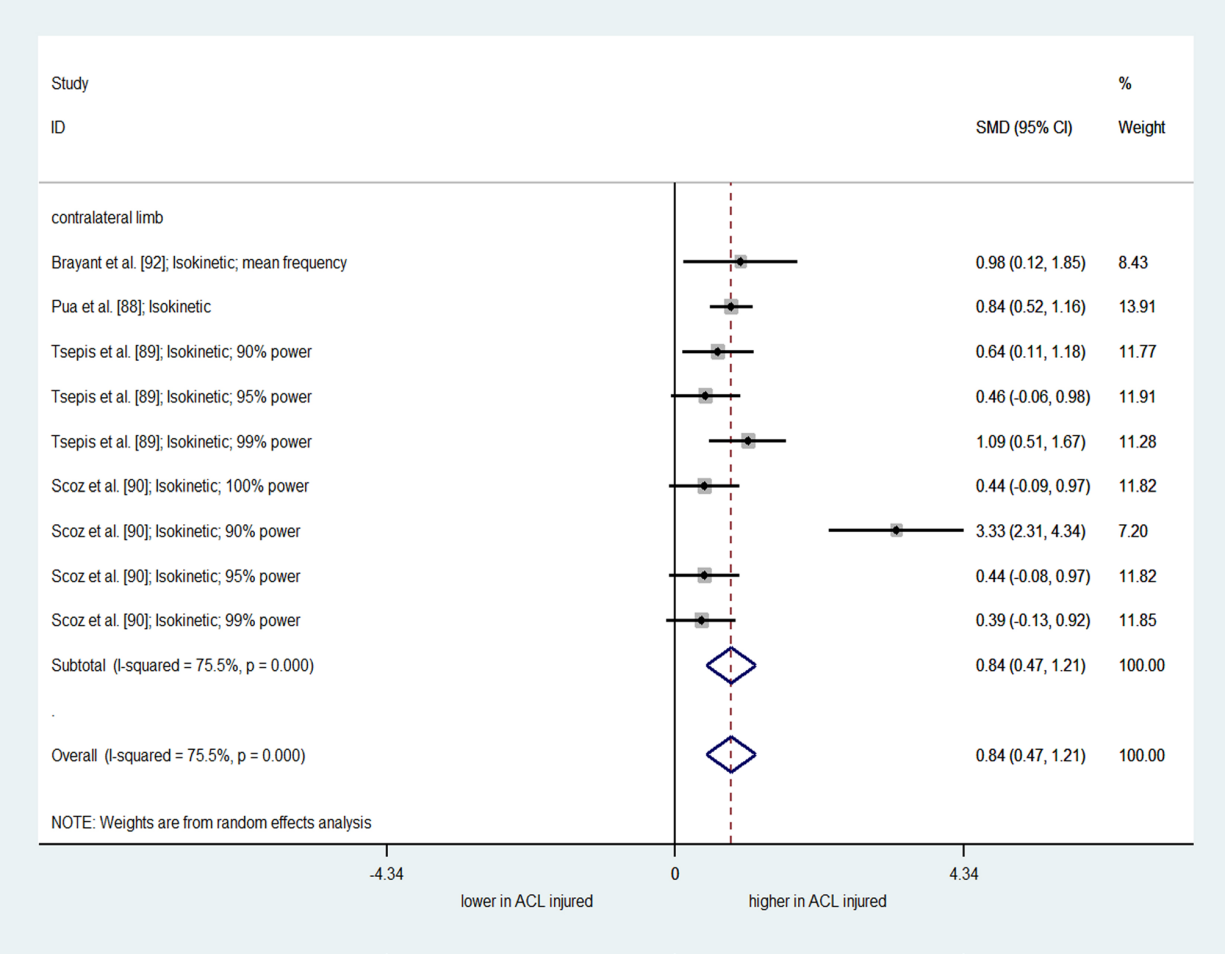
Forest plot depicting the pooled standardized mean difference (95% confidence intervals (lower limit to upper limit)) of the frequency content of quadriceps force signal between the affected limb and unaffected limb of individuals with ACL injury. SMD= Standardized mean differences. 95%CI = 95% Confidence interval. ACL= Anterior cruciate ligament

**Fig. 3:**
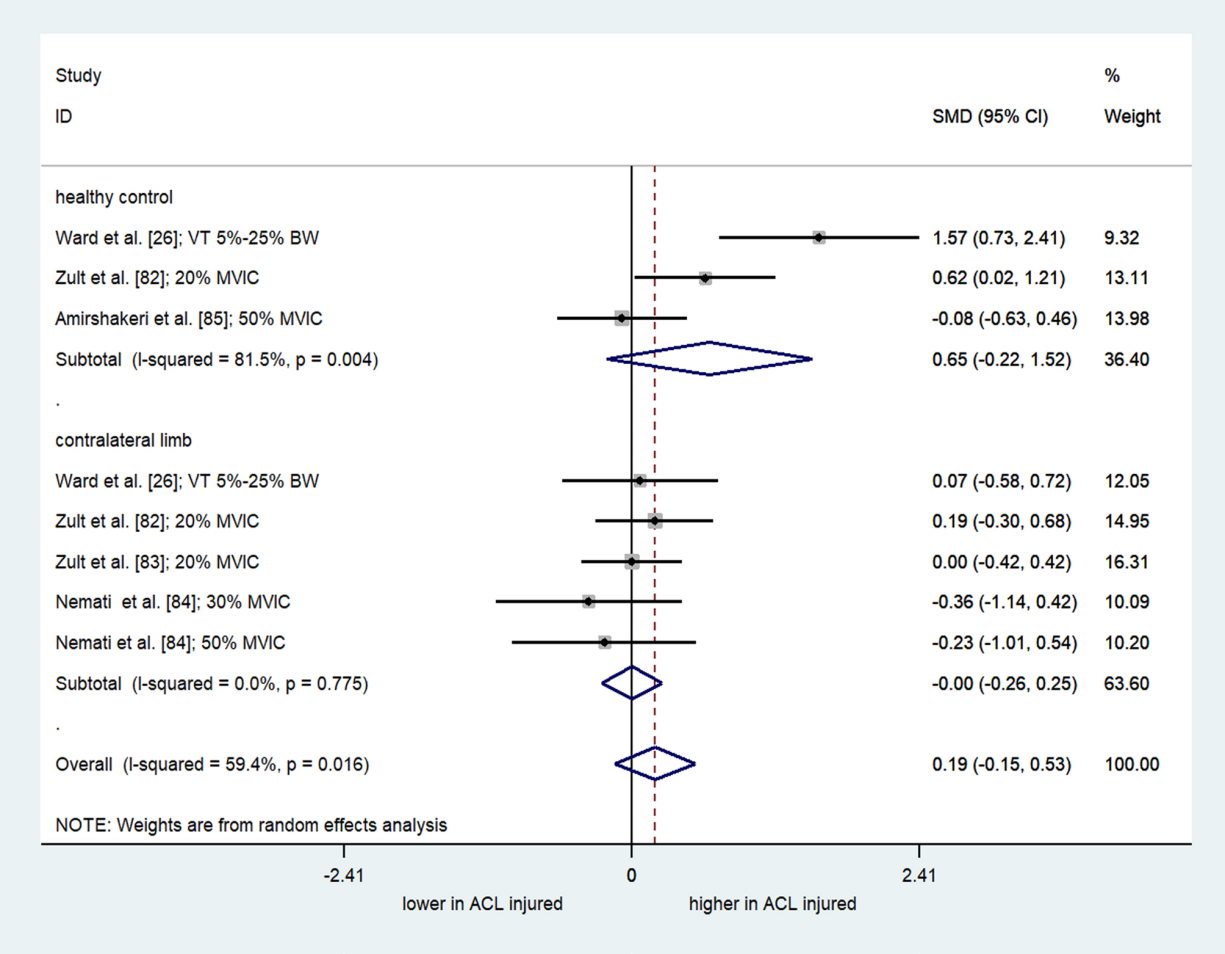
Forest plot depicting the pooled standardized mean difference (95% confidence intervals (lower limit to upper limit)) of the Root Mean Square Error of quadriceps force signal between individuals with ACL injury and healthy controls, and/or affected limb and unaffected limb of individuals with ACL injury. SMD= Standardized mean differences. 95%CI = 95% Confidence interval. ACL= Anterior cruciate ligament. VT= Variable target. BW= Body weight. MVIC: Maximum Voluntary Isometric Contraction

A meta-analysis of nine comparisons from four studies (two “high” quality [88, 90] and two “moderate” quality [89, 92]) showed a significant overall effect of ACL injury on frequency content of quadriceps torque (SMD = 0.843; 95% CI = 0.472 to 1.214; I^2^= 75.5%; τ^2^ = 0.23; Q = 32.60; *p* < 0.001) (Fig. 2). This indicated a greater frequency content of quadriceps force signal in the injured limb compared to the contralateral limb. However, considerable heterogeneity suggests that the single pooled estimate should be interpreted with caution. In contrast, a meta-analysis comprising eight comparisons from five studies (one high-quality study [83] and four moderate-quality studies [26, 82, 84, 85]), showed no significant overall effect of ACL injury on RMSE/AE of quadriceps force control (SMD = 0.19; 95% CI= -0.15 to 0.53; I^2^= 59.4%; τ^2^ = 0.13; Q = 17.24; *p* = 0.016) (Fig. 3). Subgroup analysis based on comparators revealed no differences between individuals with ACL injury and the uninjured controls (SMD = 0.653; 95%CI= -0.22 to 1.52; I^2^= 81.5%; τ^2^ = 0.47; Q = 10.81; *p* = 0.004) or contralateral limb (SMD= -0.002; 95% CI= -0.26 to 0.25; I^2^= 0.0%; τ^2^ < 0.001; Q = 1.79; *p* = 0.775). Subgroup analysis of RMSE/AE for low isometric contraction intensity (< 50% MVIC) in comparison to the unaffected contralateral limb revealed no effect (supplementary materials; SMD = 0.02; 95% CI= -0.24 to 0.30; I^2^= 0.0%; τ^2^ < 0.001; Q = 1.40; *p* = 0.704).

**Fig. 4:**
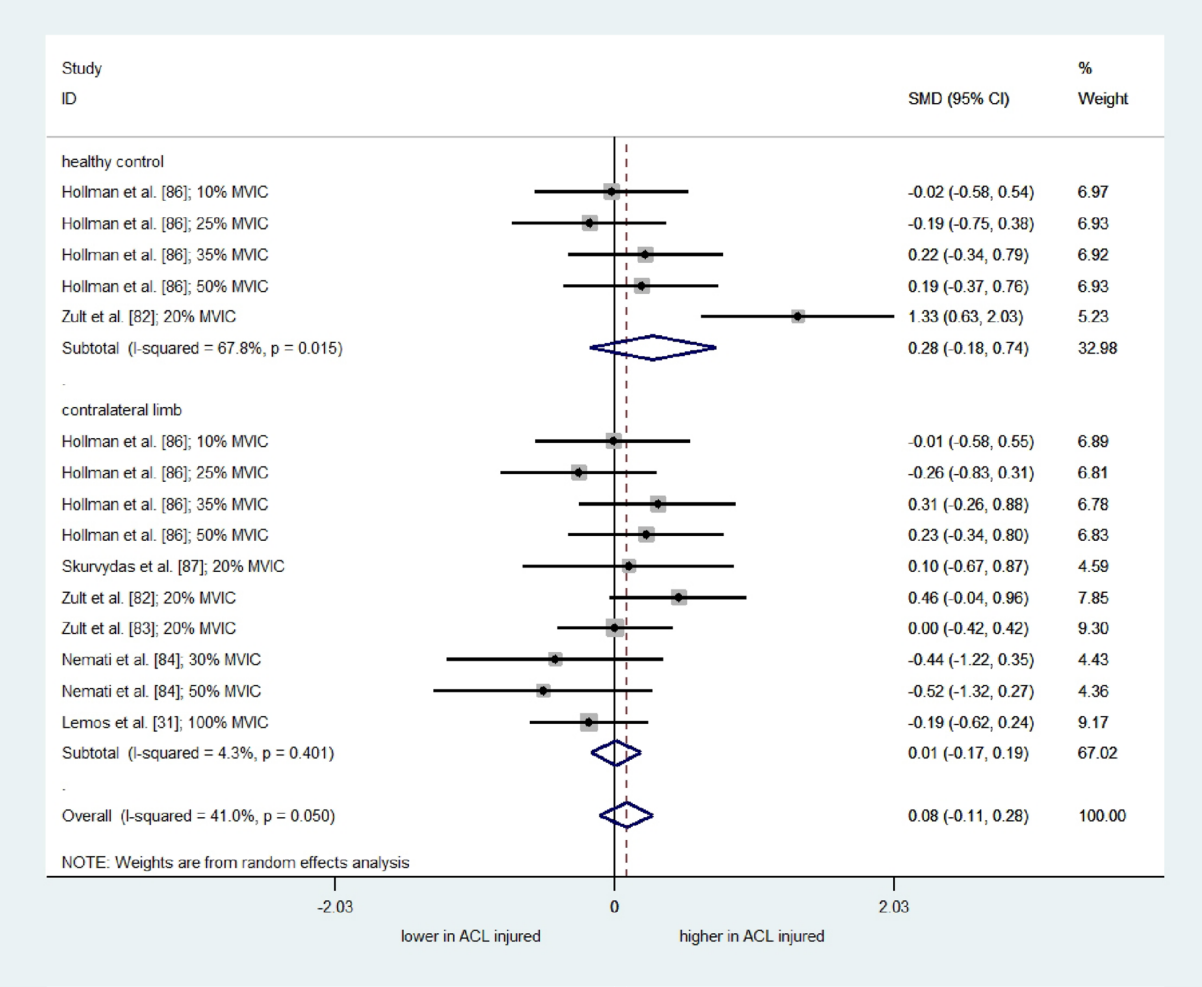
Forest plot depicting the pooled standardized mean difference (95% confidence intervals (lower limit to upper limit)) of the Coefficient of Variation of quadriceps force signal between individuals with ACL injury and healthy controls, and/or affected limb and unaffected limb of individuals with ACL injury. SMD= Standardized mean differences. 95%CI = 95% Confidence interval. ACL= Anterior cruciate ligament. MVIC: Maximum Voluntary Isometric Contraction

A separate meta-analysis of 15 comparisons from six studies (one “low” quality [86]), one “high” quality [83], and four “moderate” quality [31, 82, 84, 87]) showed no significant overall effect of ACL injury on CoV/SD of quadriceps force control (Fig. 4; SMD = 0.08; 95% CI= -0.11 to 0.28; I^2^= 41.0%; τ^2^ = 0.05; Q = 23.71; *p* = 0.050). The results of subgroup analysis, stratified by comparators, were also not significant (comparison with uninjured control group: SMD = 0.28; 95% CI= -0.18 to 0.74; I^2^= 67.8%; τ^2^ = 0.18; Q = 12.42; *p* = 0.015; comparison with the unaffected contralateral limb: SMD = 0.01; 95% CI= -0.17 to 0.19; I^2^= 4.3%; τ^2^ = 0.003; Q = 9.41; *p* = 0.401). Subgroup analysis indicated no effects for both low (< 50% MVIC) and high (≥ 50% MVIC) contraction intensities on the CoV/SD of the quadriceps force signal in individuals with ACL injuries compared to uninjured controls and the unaffected contralateral limb (supplementary materials).

Overall, these findings suggest that while ACL injury is associated with increased frequency content in the quadriceps torque signal, there are no significant differences in force fluctuations or accuracy between the injured limb and the unaffected contralateral limb or uninjured controls.

### Force Control Measures in ACL Reconstruction

Twenty-one studies compared quadriceps force control in individuals with ACLR to their contralateral unaffected limb (*n* = 4) [28, 29, 34, 80], uninjured controls (*n* = 10) [21, 22, 32, 73–79], or both uninjured controls and the unaffected contralateral limb (*n* = 7) [25, 30, 33, 71, 72, 81, 92]. Eleven studies assessed knee extensor force control using one of the CoV and/or SD measures. Out of these, seven studies found no difference either between the affected and unaffected contralateral limbs of individuals with ACLR, nor between the ACLR group and uninjured controls [30, 32, 33, 72, 73, 76, 81]. Four studies evaluated quadriceps force control at maximal effort (100% MVIC) or at 70%MVIC, all of which found higher CoV in the ACLR group than in uninjured controls, or in the affected limb of ACLR individuals than in their contralateral unaffected limb [22, 34, 75, 80]. However, Spencer et al. [80] found that a lower SD of the force signal was also observed in the affected limb compared to the unaffected contralateral limb. Since SD was not reported in other studies and CoV is a normalized SD, it was excluded from this meta-analysis. Similarly, Niederer et al. [76] reported only absolute variability, which was also excluded.

Six studies quantified force control by measuring RMSE, and four studies found greater RMSE in the ACLR group compared to uninjured controls [21, 72, 74, 79], one study [33] found no difference in normalized RMSE between ACLR group and uninjured controls, and one [71] found greater normalized RMSE in the ACLR group compared to uninjured controls, observing only at 100% MVIC (maximal effort). Among these six studies, two [21, 74] used sinusoidal targets (5%-30%), while the other four had constant targets ranging from 25% MVIC to 100% MVIC [71, 72, 79]. However, in three studies conducted by Sherman et al. [33, 71, 72], no difference in RMSE was observed between the affected limb of ACLR individuals and their contralateral unaffected limb. One study [77] measured aberration error during a force reproduction task with a target set at 50% MVIC and found no difference between the ACLR group and uninjured controls.

Two studies [25, 28] assessed the complexity of the force signal; one reported lower determinism and entropy in the force signal produced by the ACLR -affected limb during isokinetic contraction at 60°/s compared with the contralateral unaffected limb [28]. In contrast, the other one found greater approximate entropy in the force signal produced by the ACLR-affected limb during isometric contraction at maximal effort compared to both the contralateral unaffected limb and the control group [25]. Cobian et al. [29] reported a unique variable that differed from the other studies and was, therefore, excluded from the meta-analysis.

Frequency-domain analyses indicated altered spectral characteristics post-ACLR. One study [92] found that the mean instantaneous frequency of the extension torque produced by the ACLR-affected limb during isokinetic contraction at 180°/s was higher compared to both the contralateral unaffected limb and the uninjured control group. Hunt et al. [32] found a greater peak power frequency in the ACLR group compared to uninjured controls. Also, one study [78] assessed quadriceps force control during isokinetic contraction at 60°/s and observed higher ​​Orthogonal Daubechies values and biorthogonal wavelets in the ACLR group compared to the healthy control group.

### Results of Meta-Analyses on ACL Reconstruction

**Fig. 5:**
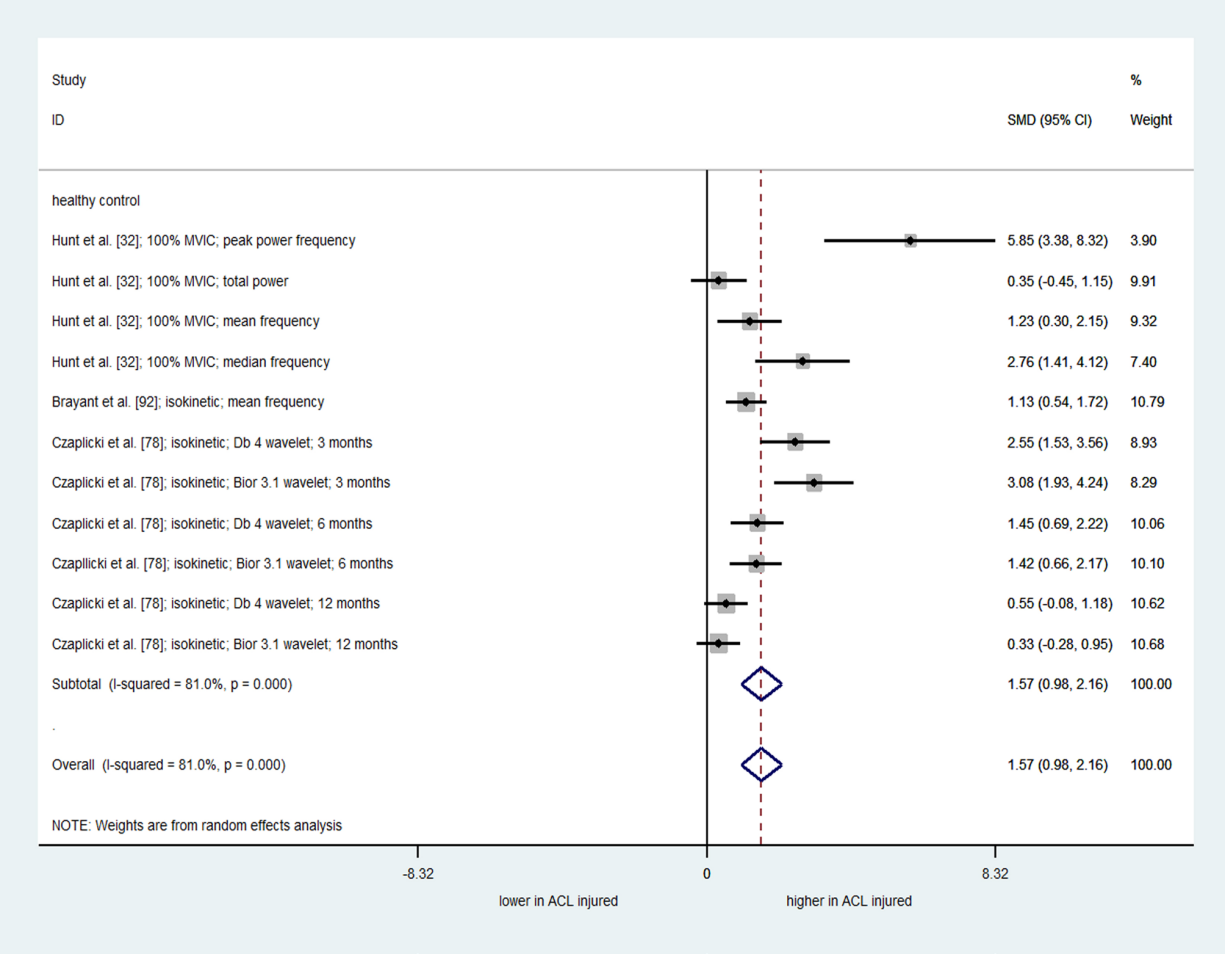
Forest plot depicting the pooled standardized mean difference (95% confidence intervals (lower limit to upper limit)) of the frequency content of quadriceps force signal between individuals with ACL reconstruction and healthy controls. SMD= Standardized mean differences. 95%CI = 95% Confidence interval. ACL= Anterior cruciate ligament. MVIC: Maximum Voluntary Isometric Contraction. Db= Daubechies. Bior: biorthogonal

A meta-analysis of eleven comparisons from three studies with moderate quality [32, 78, 92] showed a significant overall effect of ACLR on frequency content of quadriceps torque (SMD = 1.57; 95% CI = 0.98 to 2.16; I^2^= 81.0%; τ^2^ = 0.75; Q = 52.76; *p* < 0.001) (Fig. 5). This result implied a greater frequency content of the force signal in individuals with ACLR compared to the uninjured control group. However, considerable heterogeneity suggests that effects may vary by participants’ individual characteristics, such as kinesiophobia or graft type.

**Fig. 6:**
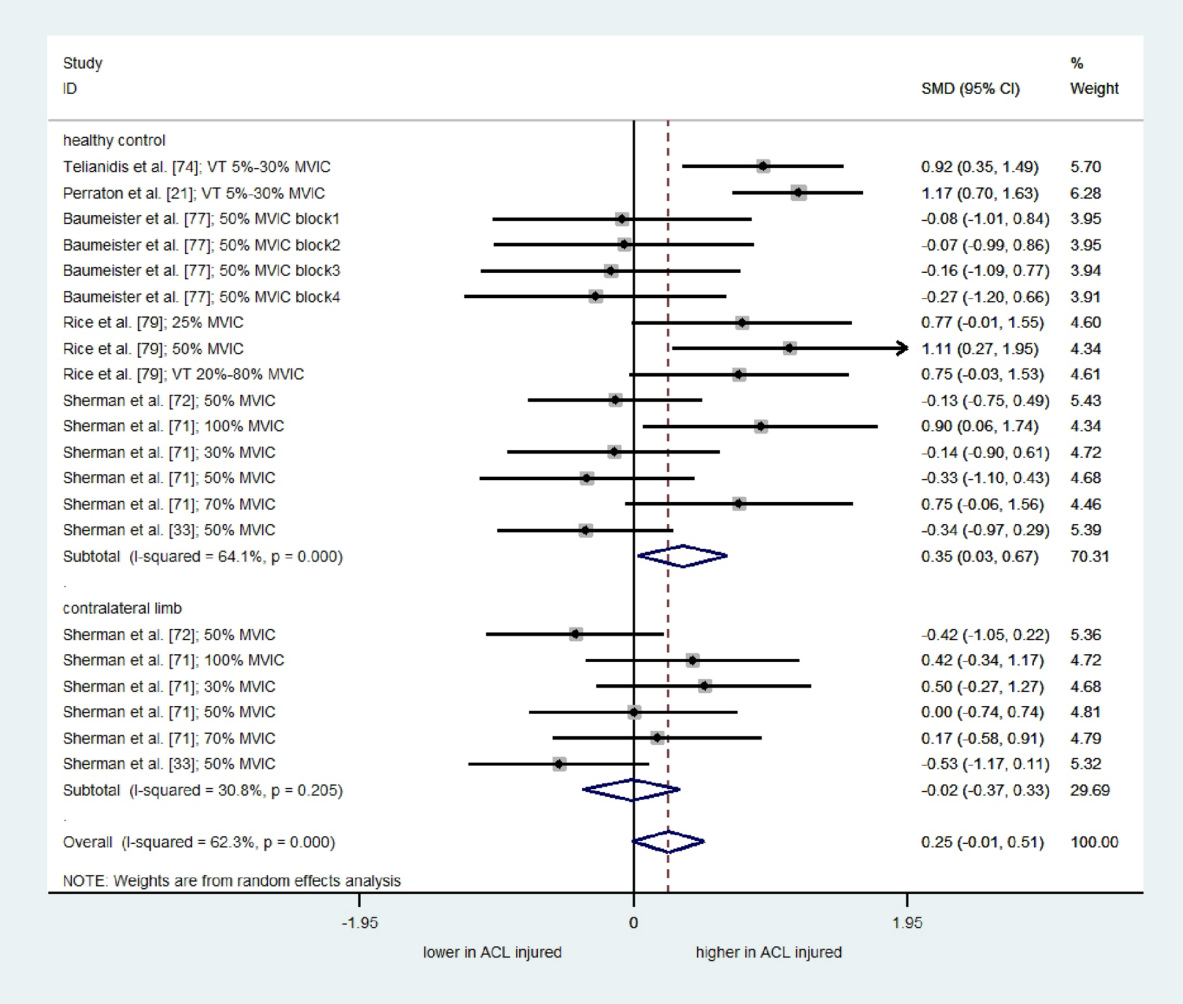
Forest plot depicting the pooled standardized mean difference (95% confidence intervals (lower limit to upper limit)) of the Root Mean Square Error of quadriceps force signal between individuals with ACL reconstruction and healthy controls, and/or affected limb and unaffected limb of individuals with ACL reconstruction. SMD= Standardized mean differences. 95%CI = 95% Confidence interval. ACL= Anterior cruciate ligament. MVIC: Maximum Voluntary Isometric Contraction

Twenty-one comparisons from seven moderate-quality studies [21, 71, 72, 74, 77, 79], showed no significant overall effect of ACLR on the RMSE/AE of quadriceps force (SMD = 0.25; 95% CI= -0.01 to 0.51; I^2^= 62.3%; τ^2^ = 0.22; Q = 53.05; *p* < 0.001) (Fig. 6). The moderate heterogeneity may also reflect differences in force control tasks (sinusoidal target versus constant target). The results of subgroup analysis indicate that the ACLR group showed greater RMSE/AE compared to the uninjured control group (SMD = 0.35; 95%CI = 0.03 to 0.67; I^2^= 64.1%; τ^2^ = 0.24; Q = 39.04; *p* < 0.001), suggesting impaired force control in people with ACLR compared to the uninjured control group. However, no significant differences were observed between the involved limb of people with ACLR and their contralateral unaffected limb (SMD= -0.02; 95%CI= -0.37 to 0.33; I^2^= 30.8%; τ^2^ = 0.05; Q = 7.22; *p* = 0.205). In terms of RMSE/AE of the quadriceps force signal, subgroup analysis showed a moderate effect for low isometric contraction intensities (< 50% MVIC), indicating lower accuracy and impaired force control in individuals with ACLR compared to uninjured controls. However, it should be noted that two of the four studies in this category used a sinusoidal target [54, 74].

**Fig. 7:**
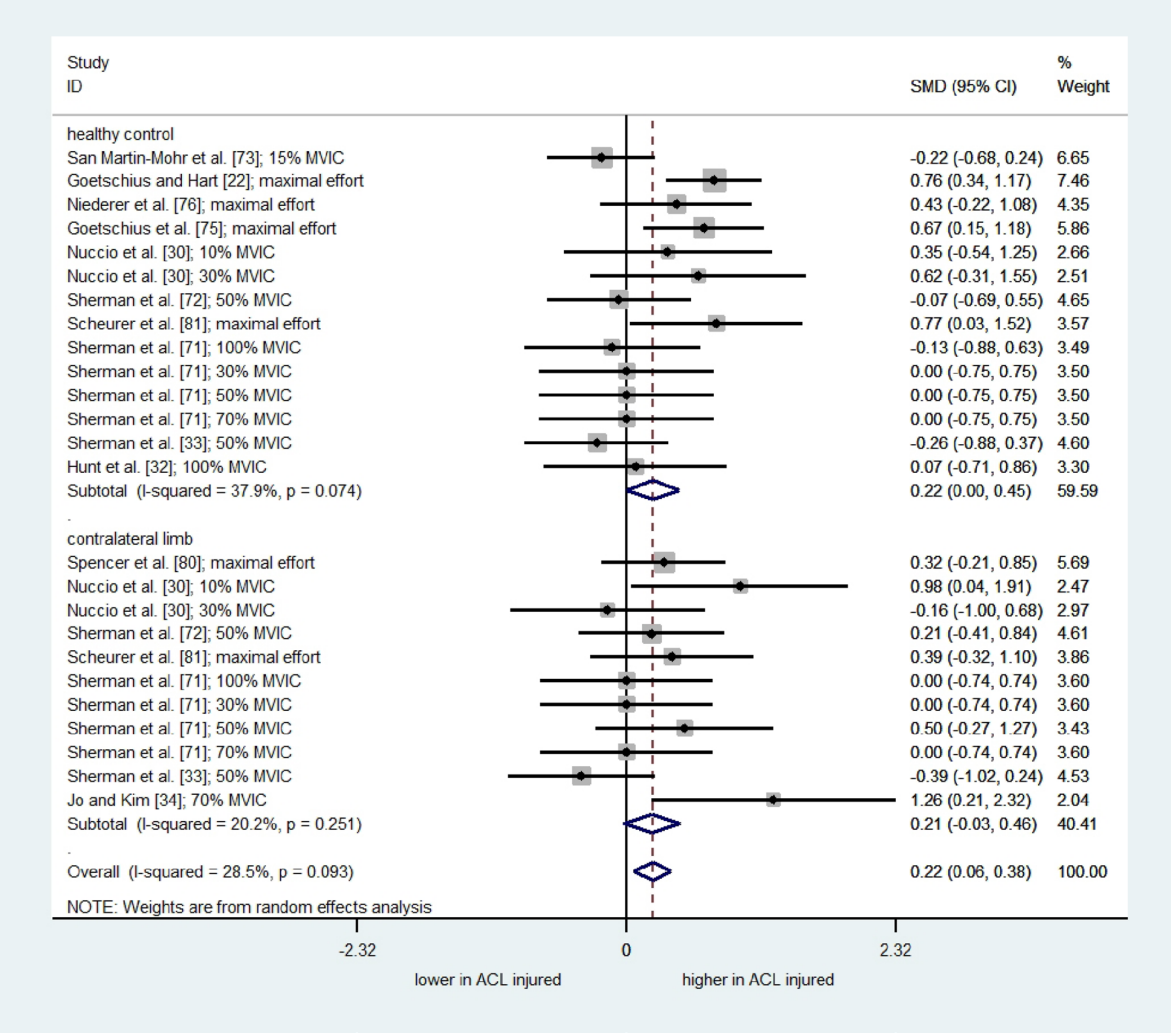
Forest plot depicting the pooled standardized mean difference (95% confidence intervals (lower limit to upper limit)) of the Coefficient of Variation of quadriceps force signal between individuals with ACL reconstruction and healthy controls, and/or affected limb and unaffected limb of individuals with ACL reconstruction. SMD= Standardized mean differences. 95%CI = 95% Confidence interval. ACL= Anterior cruciate ligament. MVIC: Maximum Voluntary Isometric Contraction

Similarly, the result of a meta-analysis of 25 comparisons from twelve studies (one low quality [73] and 11 moderate quality [22, 30, 32–34, 71, 72, 75, 76, 80, 81]) revealed a significant overall effect of ACLR on the CoV/SD of force signal (SMD = 0.22; 95% CI = 0.06 to 0.38; I^2^= 28.5%; τ^2^ = 0.046; Q = 33.56; *p* = 0.093) (Fig. 7). Subgroup analysis, stratified by comparators, showed a significant difference in the CoV of force signal between the ACLR and healthy controls (SMD = 0.22; 95% CI = 0.001 to 0.45; I^2^= 37.9%; τ^2^ = 0.06; Q = 20.94; *p* = 0.074). However, no significant difference was found between the involved limb compared to their contralateral uninvolved limb (SMD = 0.21; 95% CI= -0.03 to 0.46; I^2^= 20.2%; τ^2^ = 0.03; Q = 12.54; *p* = 0.251). Subgroup analysis for isometric contraction intensities revealed a small effect for high intensities (≥ 50% MVIC), suggesting greater force fluctuations in ACLR relative to uninjured controls (Supplementary materials). Overall, these findings indicate impaired force steadiness, with greater force fluctuations and reduced accuracy in individuals with ACLR compared to uninjured controls.

### Sensitivity Analyses

In ACL injury, excluding single outlier studies (Zult et al. [82]; Ward et al. [26]) for both CoV-based and RMSE-based outcomes eliminated statistical heterogeneity while null effect sizes with tighter confidence intervals were maintained. This pattern indicates that the heterogeneity was due to these outliers. After exclusion, all heterogeneity disappeared, and the pooled estimates moved closer to zero, suggesting robust null conclusions. The remaining studies consistently support a lack of meaningful effect. For the frequency content-based outcome, a single condition from one study [90] disproportionately influenced the large effect size and heterogeneity. By excluding that study, substantial heterogeneity was completely resolved, and the clinical interpretation shifted from a large effect (SMD > 0.8) to a moderate effect (SMD = 0.66).

In ACLR, sensitivity analyses showed that excluding any single study did not affect the overall results of meta-analyses for CoV, RMSE, or frequency content outcomes. Excluding low-quality studies, meta-analyses of frequency content-based outcomes in ACL injury were limited to two high-quality studies [88, 90]. When moderate-quality studies were excluded [89, 92], both the effect size and statistical heterogeneity increased (SMD = 0.95; 95% CI = 0.31 to 1.60; I² = 86.5%; τ² = 0.43; Q = 29.63; *p* < 0.001). This suggests that high-quality studies indicate a stronger effect but also greater heterogeneity, reinforcing the impairment of quadriceps force signal frequency content in individuals with ACL injuries. The meta-analysis of CoV-based outcomes in ACL injury indicated that excluding eight comparisons from a poor-quality study [86] slightly increased the point estimate but remained negligible and non-significant (SMD = 0.14; 95% CI = -0.37 to 0.65; I² = 74.5%; τ² = 0.29; Q = 19.64; *p* = 0.001). This exclusion significantly increased heterogeneity, suggesting misleading consistency among studies. Furthermore, removing this study from the CoV analysis of force signal in ACLR did not change the effect size or heterogeneity (SMD = 0.25; 95% CI = 0.09 to 0.42; I² = 22.3%). Overall, the sensitivity analysis confirmed the robustness of our findings (detailed results are provided in the supplementary materials).

### Association of Force Control Measures and Physical Function

Only five studies [21, 22, 31, 88, 92] have investigated associations between quadriceps force control and self-reported knee function or functional performance in individuals with ACL injury or ACLR. Two studies [88, 92] examined the relationship between torque frequency produced during concentric contraction and functional performance (hop test). The first study [88] found that greater quadriceps force steadiness was associated with better hop performance in individuals with ACL injury. In contrast, the second study [92] reported inconsistent results, indicating that impaired force steadiness was associated with faster performance on the single-limb hopping test in individuals with ACL injury and/or ACLR.

Another study [31] showed a moderate negative correlation between the single hop test and the sample entropy of the force signal produced during isometric contraction at the maximal level in individuals with ACL injury. Two other studies [21, 22] investigated the relationship between quadriceps force control during isometric contraction and self-reported knee function in individuals with ACLR. The findings indicated that less accurate quadriceps force output (greater RMSE) or higher torque variability was associated with poorer knee joint function. These studies used self-reported questionnaires, such as the Cincinnati Knee Rating Scale (CKRS) and the International Knee Documentation Committee (IKDC), to assess knee function. However, Perraton et al. [21] included three single-leg hop tests, in addition to self-reported questionnaires, to evaluate functional performance.

## Discussion

The current systematic review synthesized evidence on changes in quadriceps force control in individuals with ACL injury and/or ACLR. We also explored the relationship between force control and functional performance and/or self-reported knee function. The main findings of this meta-analysis revealed no significant differences in the magnitude of force fluctuation (measured by CoV/SD) or accuracy of force output (RMSE/AE) between the affected limbs of individuals with ACL injury, their contralateral unaffected limbs, or uninjured controls. However, studies that decomposed force signals into their underlying frequency components, using techniques such as the Fast Fourier and/or wavelet transform, showed force control deficits in the affected limbs of individuals with ACL injury compared to their contralateral unaffected limb. In addition, data synthesis demonstrated impaired force accuracy (i.e., a greater deviation of force output from the target force) as well as greater force signal variability and higher frequency content of force signal in individuals with ACLR relative to healthy controls, suggesting impaired quadriceps force control following reconstruction. Furthermore, findings of this review indicated that deficient quadriceps force control adversely impacts functional capacity in patients who have sustained an ACL injury or undergone surgical reconstruction.

### ACL Injury

This review found that the accuracy of quadriceps force signals in the affected limb of individuals with ACL injury did not differ from their contralateral unaffected limb or from uninjured controls. Notably, one study [26] reported a large effect size, possibly due to the unique method of the force-matching task. This study employed a sinusoidal force target (5%-25% of body weight), whereas other studies used a constant target force to assess force control [82–85]. Also, the magnitude of quadriceps force fluctuation (CoV/SD) in the affected limb of ACL-injured individuals did not differ from either their contralateral unaffected limb or uninjured controls. Meta-analysis of frequency-based outcomes showed a large effect size, indicating higher frequency levels of quadriceps force in the affected limb of ACL-injured individual compared to their contralateral unaffected limb. These studies employed isokinetic contractions [88–90, 92]. While other studies on force variability and accuracy have assessed force control tasks under isometric contractions, which produce more consistent force than isokinetic contractions [93], this may partially explain these differences.

Impaired force control in individuals with ACL injuries may stem from adopting protective stiffening strategies or kinesiophobia and fear of re-injury [94–97]. These people often adopt guarding strategies, like increased hamstring co-contraction, to stabilize the joint by enhancing stiffness and reducing shear forces [74]. While mild co-contraction can improve stability, excessive co-contraction in unstable knees can increase torque variability and disrupt motor control due to signal-dependent noise [98–100]. Proprioceptive deficits and inaccurate sensory information may also reduce the CNS’s ability to generate and regulate appropriate muscle force [101].

Three studies, Lemos et al. [31], Skurvydas et al. [87], and Hollman et al. [86] reported insignificant differences in the magnitude of force fluctuations (measured by CoV) between the affected limb and the contralateral unaffected limb or uninjured controls. However, these three studies reported increased sample entropy, reduced permutation entropy, and increased fractal scaling exponent when assessing non-linear measures of the force signal, respectively. Reduced entropy indicates more predictable and regular quadriceps force output, while an increased fractal exponent suggests a loss of complexity of the force signal [102, 103]. This implies that conventional linear magnitude-based measures are not sensitive enough to identify underlying deficits in sensorimotor control. It is also worth noting that the target force levels in the studies ranged from 5% to 100% MVIC. It is hypothesized that higher force targets may not effectively detect force control deficits [13, 91]. For example, Johnson et al. [91] found no differences in sample entropy at 100% MVIC, whereas Hollman et al. [86] observed significant differences in the fractal scaling exponent at 25% and 35% MVIC, but not at 50% MVIC. Given that most daily activities occur at submaximal levels [104, 105], assessing force control during submaximal contractions is essential.

Due to methodological variability and the limited number of studies, subgroup analysis based on elapsed time since surgery was not feasible. For example, impaired force steadiness was observed in the first weeks after injury in Pua et al. [88] and even years later in Bryant et al. [92]. These inconsistent findings, where some studies report no quadriceps force steadiness deficits in the acute phase while others identify long-term deficits, suggest that a strict timeline for neuromuscular recovery cannot be defined [106]. This variability indicates individual differences in neural adaptation may be a contributing factor. Specifically, those who demonstrate normalized force steadiness early may represent “potential copers” who develop effective compensatory strategies. In contrast, those with prolonged deficits likely represent “non-copers,” whose sensorimotor systems struggle to maintain stable joint control, even years post-injury [107].

### ACL Reconstruction

Individuals with ACLR exhibited significantly higher frequency content, lower quadriceps force accuracy (RMSE/AE) and increased force variability (CoV) compared to uninjured controls, indicating ongoing neuromuscular deficits. However, both meta-analyses regarding force signal accuracy and variability revealed a small effect size and its confidence interval encompassed several interpretive regions: trivial, small, and moderate. This indicates that these findings are inconclusive. Furthermore, the absence of difference between the affected and unaffected limbs in individuals with ACLR supports previous research on neuromuscular adaptation in the unaffected contralateral side [108, 109]. A plausible mechanism to explain the deficits observed in the quadriceps following ACLR can be attributed to alterations in motor unit behavior [110]. Nuccio et al. [52] and Sherman et al. [71] reported decreased neural drive and inefficient motor unit modulation following ACLR may contribute to persistent quadriceps strength deficits.

Impaired quadriceps force control, shown by high-frequency fluctuations in the injured limb compared to the uninjured limb, is linked to altered sensory inputs from damaged ACL mechanoreceptors and reflex-mediated suppression of alpha-motoneuron excitability [12, 111]. In other words, this altered motor inhibition results from ligament and joint damage, pain, and effusion [111, 112]. Although spinal reflex excitability remains low for months after reconstruction, surgical stabilization and pain reduction may restore full alpha-motoneuron recruitment, suggesting that decreased reflex excitability might not significantly affect post-surgical force steadiness [111, 113, 114]. Impaired force control in ACLR, observed in both linear metrics and frequency analyses, suggests that restoring mechanical stability alone does not fully normalize central motor drive and cortical descending pathways [113]. Previous studies using transcranial magnetic stimulation have reported deficits in quadriceps corticospinal excitability in ACLR, characterized by higher cortico-motor thresholds and reduced motor evoked potential magnitudes [14, 16, 81]. Furthermore, maladaptive neuroplasticity and cortical reorganization in ACLR may further reduce specific descending drive from the primary motor cortex, resulting in poorly controlled force output and increased fluctuations [115].

Two studies assessed force control using non-linear metrics [25, 28]. Chaney et al. [28] observed lower entropy compared to the contralateral side, indicating a less adaptable and flexible neuromuscular system. In contrast, Bodkin et al. [25] reported higher entropy values, indicating a noisy and unstable system. The theory of optimal movement variability suggests that the regularity of the force signals observed in healthy individuals indicates a well-functioning and efficient sensorimotor system. Any deviation from this optimal state, toward less regularity or more regularity, indicates an impaired neuromuscular system [116].

Subgroup analysis based on the time since ACLR surgery was not feasible. Neuromuscular control often normalizes over a prolonged period, taking up to 24 months [117]. For example, Czaplicki et al. [78] found force steadiness deficits at 3 and 6 months resolved by 12 months post-surgery. In contrast, some studies have reported significant differences persisting for 3 years or more [22, 75, 81]. This discrepancy may result from factors such as late-stage neuromuscular re-education intensity, persistent kinesiophobia, and graft type.

### Impact of Contraction Intensity

Our subgroup analysis showed greater RMSE in individuals with ACLR compared to uninjured controls at low contraction intensity, with 2 of 4 comparisons using a sinusoidal target [54, 74]. Individuals with ACLR also exhibited a higher CoV at greater contraction intensities. These findings suggest that force steadiness depends on contraction intensity, reflecting fundamental neuromuscular recruitment principles, as RMSE scales with force magnitude [118]. At higher contraction intensities (> 50–70% MVIC), greater motor unit recruitment and larger muscle forces amplify fluctuations [118, 119], making group differences in absolute force instability statistically detectable. Conversely, small fluctuations at low forces (< 20% MVIC) may mask subtle impairments when measured by RMSE alone.

This limitation is addressed by dynamic force-tracking tasks (e.g., sinusoidal targets), where RMSE measures tracking accuracy, integrating measures of visuomotor processing, predictive control, real-time error correction, and proprioceptive feedback [74]. This method is highly sensitive to neural deficits at submaximal levels (5–30% MVIC) [74]. In ACL injury and ACLR, proprioceptive loss and disrupted sensorimotor integration impair fine force modulation, leading to jerky contractions and increased RMSE [26]. Additionally, reliance on slower visual feedback to compensate for impaired proprioception increases errors at low force levels [120], as seen in 2 of 4 studies using sinusoidal targets.

CoV, which normalizes the standard deviation of force to its mean, making it sensitive to fluctuations [118]; however, low mean values at low forces result in high CoV values [121]. Deficits in ACLR are therefore most evident at low intensities, where precise rate coding of low-threshold motor units is affected by arthrogenic inhibition and altered sensory input [118]. However, our findings show higher CoV at 100% MVIC may reflect maladaptive compensation strategies related to fear of re-injury. Therefore, CoV at maximal effort assesses both steadiness and the quality of maximal voluntary activation.

### Force Control Association with Physical Functional Performance

Both studies, Pua et al. [88] and Bryant et al. [92], examined the frequency content of force signals during isokinetic contractions; however, they reported contradictory results regarding the relationship between force steadiness and hop performance. Pua et al. [88] found that better force steadiness was associated with enhanced hop performance, whereas Bryant et al. [92] reported the opposite; impaired force steadiness was correlated with better hop performance. These discrepancies may be partly explained by differences in contraction velocity. At a velocity of 60°/s, impaired steadiness likely reflects better recruitment of slow-speed motor units, which helps in controlled landings and hop distance [122]. Conversely, at a velocity ≥ 180°/s, slight force irregularities may reflect adaptations for rapid force production, facilitating quicker hop times.

Importantly, impaired steadiness during isometric contractions has been associated with poorer patient-reported outcomes (IKDC/CKRS), underscoring the role of quadriceps force control in perceived stability during daily activities. These inconsistencies highlight the limitations of non-weight-bearing force steadiness assessment in capturing the complex neuromuscular strategies employed during functional movements, where compensatory mechanisms can potentially mask underlying deficits.

## Strengths and Limitations

This systematic review and meta-analysis has several strengths, including the prospective registration in PROSPERO and adherence to the PRISMA checklist. However, some limitations should be acknowledged. The exclusion of grey literature may have introduced publication bias. Moreover, a limited number of high-quality studies available for analysis may reduce the robustness of the findings. Additionally, inherent limitations of the included observational studies, such as heterogeneity in participants’ characteristics (time since injury/surgery, surgical technique), prevent causal inferences and may introduce confounding. Therefore, these limitations should be considered when interpreting and applying the findings of this review.

### Future Research

To further enhance our understanding in this field, high-quality longitudinal studies are needed to examine the quality of force control following ACL injury and reconstruction surgeries. Further research should also investigate the relationship between quadriceps force control and functional outcomes to provide a more comprehensive assessment of ACL recovery progression. Potential contributing factors, particularly time since injury or surgery, should also be explored for their impact on quadriceps force control adaptations. Also, applying non-linear analysis of force signals may provide deeper insights into neuromuscular control mechanisms following ACL injury, offering a promising direction for future research.

### Clinical Implications

Frequency content analysis reveals differences between injured and contralateral limbs, whereas linear metrics (CoV, RMSE) may overestimate recovery or miss asymmetries. Non-linear measures can provide insights into neural mechanisms assisting targeted rehabilitation, but more high-quality studies are needed. Post-reconstruction comparisons with the contralateral limb may underestimate impairments due to bilateral neuromuscular adaptations; therefore, including healthy control data is essential for identifying persistent deficits. Relying only on linear measures (CoV, RMSE) can overlook important clinically relevant deficits. Including non-linear signal analysis offers a comprehensive assessment to capture complexity-related changes in motor control. Additionally, the association between quadriceps force steadiness and functional capacity underscores the importance of incorporating force control training into rehabilitation programs to improve neuromuscular control, even in patients with symmetrical strength.

## Conclusion

The findings of this systematic review and meta-analysis suggest that frequency content analyses reliably detect impaired quadriceps force control in the injured limb of individuals with ACL injury, highlighting deficits that linear measures may miss. Although ACLR improves mechanical joint stability, it does not fully restore neuromuscular function. Persistent abnormalities in force control are clinically meaningful and can diminish physical functioning in both ACL injury and ACLR populations. Therefore, clinicians should integrate targeted neuromuscular training into rehabilitation programs to enhance quadriceps control and improve functional outcomes. Including both linear and non-linear signal analyses, as well as healthy control comparisons, can better identify deficits and guide individualized treatment.

## Supplementary Information

Below is the link to the electronic supplementary material.

Supplementary Material 1.

Supplementary Material 2.

Supplementary Material 3. Forest plot depicting the pooled standardized mean difference (95% confidence intervals (lower limit to upper limit)) of the Root Mean Square Error of quadriceps force signal between affected limb and unaffected limb of individuals with ACL injury, in low intensity isometric contractions (<50% MVIC). SMD= Standardized mean differences. 95%CI= 95% Confidence interval. ACL= Anterior cruciate ligament. MVIC: Maximum Voluntary Isometric Contraction. VT= Variable target. BW= Body weight.

Supplementary Material 4. Forest plot depicting the pooled standardized mean difference (95% confidence intervals (lower limit to upper limit)) of the Coefficient of Variation of quadriceps force signal between individuals with ACL injury and healthy controls, in low intensity isometric contractions (<50% MVIC). SMD= Standardized mean differences. 95%CI= 95% Confidence interval. ACL= Anterior cruciate ligament. MVIC: Maximum Voluntary Isometric Contraction.

Supplementary Material 5. Forest plot depicting the pooled standardized mean difference (95% confidence intervals (lower limit to upper limit)) of the Coefficient of Variation of quadriceps force signal between affected limb and unaffected limb of individuals with ACL injury, in 1: low (<50% MVIC) and 2: high (≥50% MVIC) intensity isometric contractions. SMD= Standardized mean differences. 95%CI= 95% Confidence interval. ACL= Anterior cruciate ligament. MVIC: Maximum Voluntary Isometric Contraction.

Supplementary Material 6. Forest plot depicting the pooled standardized mean difference (95% confidence intervals (lower limit to upper limit)) of the Root Mean Square Error of quadriceps force signal between individuals with ACL reconstruction and healthy controls, in 1: low (<50% MVIC) and 2: high (≥50% MVIC) intensity isometric contractions. SMD= Standardized mean differences. 95%CI= 95% Confidence interval. ACL= Anterior cruciate ligament. MVIC: Maximum Voluntary Isometric Contraction.

Supplementary Material 7. Forest plot depicting the pooled standardized mean difference (95% confidence intervals (lower limit to upper limit)) of the Root Mean Square Error of quadriceps force signal between affected limb and unaffected limb of individuals with ACL reconstruction, in high (≥50% MVIC) intensity isometric contractions. SMD= Standardized mean differences. 95%CI= 95% Confidence interval. ACL= Anterior cruciate ligament. MVIC: Maximum Voluntary Isometric Contraction.

Supplementary Material 8. Forest plot depicting the pooled standardized mean difference (95% confidence intervals (lower limit to upper limit)) of the Coefficient of Variation of quadriceps force signal between individuals with ACL reconstruction and healthy controls, in 1: low (<50% MVIC) and 2: high (≥50% MVIC) intensity isometric contractions. SMD= Standardized mean differences. 95%CI= 95% Confidence interval. ACL= Anterior cruciate ligament. MVIC: Maximum Voluntary Isometric Contraction.

Supplementary Material 9. Forest plot depicting the pooled standardized mean difference (95% confidence intervals (lower limit to upper limit)) of the Coefficient of Variation of quadriceps force signal between affected limb and unaffected limb of individuals with ACL reconstruction, in 1: low (<50% MVIC) and 2: high (≥50% MVIC) intensity isometric contractions. SMD= Standardized mean differences. 95%CI= 95% Confidence interval. ACL= Anterior cruciate ligament. MVIC: Maximum Voluntary Isometric Contraction.

## Data Availability

The studies included in this systematic review are listed in the References section and can be accessed through various databases.

## Abbreviations

ACL: Anterior cruciate ligament
ACLR: Anterior cruciate ligament reconstruction
CNS: Central nervous system
SD: Standard deviations
SE: Standard Error
SMD: Standardized mean difference
RMSE: Root mean squared error
AE: Absolute error
CoV: Coefficient of variation
BPT: Bone-patellar tendon-bone
HT: Hamstring tendon graft
MVIC: Maximum Voluntary Isometric Contraction
95%CI: 95% Confidence interval
VT: Variable target
BW: Body weight
