# Supplementary material for: Changes in Quadriceps Force Control and Torque Quality Following Anterior Cruciate Ligament Injury and Reconstruction: Associations with Functional Performance—A Systematic Review and Meta-Analysis
Source: Sports Med Open. 2026 May 11;12:54. doi: 10.1186/s40798-026-00999-x (PMC13158337; doi:10.1186/s40798-026-00999-x)
Supplement: Supplementary file 1 — Supplementary Material 1. [file 40798_2026_999_MOESM1_ESM.docx]

Supplementary 1. Search strategies in databases:

| **PubMed:**  ((("force control"[Title/Abstract:~5]) OR ("force modulation"[Title/Abstract:~5]) OR ("force fluctuation"[Title/Abstract:~5]) OR ("force variability"[Title/Abstract:~5]) OR ("force steadiness"[Title/Abstract:~5]) OR ("torque control"[Title/Abstract:~5]) OR ("torque quality"[Title/Abstract:~5]) OR ("torque variability"[Title/Abstract:~5]) OR ("torque-time curve"[Title/Abstract:~0]) OR ("force sense"[Title/Abstract:~5]) OR ("force perception"[Title/Abstract:~5]) OR ("force accuracy"[Title/Abstract:~5]) OR ("muscle force"[Title/Abstract:~0]) OR ("quadriceps force"[Title/Abstract:~5])) AND (("Knee Injuries"[Mesh] OR "Anterior Cruciate Ligament"[Mesh] OR "Anterior Cruciate Ligament Injuries"[Mesh] OR "Anterior Cruciate Ligament Reconstruction"[Mesh]) OR ("anterior cruciate ligament"[Title/Abstract]) OR ("anterior cruciate ligament reconstruction"[Title/Abstract]) OR (ACL[Title/Abstract]) OR (ACLR[Title/Abstract]) OR ("ACL reconstruct*"[Title/Abstract]) OR ("ACL injur*"[Title/Abstract]) OR ("ACL deficien*"[Title/Abstract]) OR ("ACL rupture"[Title/Abstract]) OR ("ACL tear"[Title/Abstract]) OR ("Knee injur*"[Title/Abstract]))) |
| --- |
| **Web of Science:**  **"anterior cruciate ligament"** (Topic) or **"anterior cruciate ligament reconstruction"** (Topic) or **ACL** (Topic) or **ACLR** (Topic) or **"ACL reconstruct*"** (Topic) or **"ACL injur*"** (Topic) or **"ACL deficien*"** (Topic) or **"ACL rupture"** (Topic) or **"ACL tear"** (Topic) or **"Knee injur*"** (Topic)  **force NEAR/5 control** (Topic) or **force NEAR/5 modulation** (Topic) or **force NEAR/5 fluctuation** (Topic) or **force NEAR/5 variability** (Topic) or **force NEAR/5 steadiness** (Topic) or **torque NEAR/5 control** (Topic) or **torque NEAR/5 quality** (Topic) or **torque NEAR/5 variability** (Topic) or **"torque-time curve"** (Topic) or **force NEAR/5 sense** (Topic) or **force NEAR/5 perception** (Topic) or **force NEAR/5 accuracy** (Topic) or **muscle NEAR/0 force** (Topic) or **quadriceps NEAR/5 force** (Topic)  **#2 AND #1** |
| **Scopus:**  ( ( TITLE-ABS-KEY ( "anterior cruciate ligament" ) OR TITLE-ABS-KEY ( "anterior cruciate ligament reconstruction" ) OR TITLE-ABS-KEY ( acl ) OR TITLE-ABS-KEY ( aclr ) OR TITLE-ABS-KEY ( "ACL reconstruct*" ) OR TITLE-ABS-KEY ( "ACL injur*" ) OR TITLE-ABS-KEY ( "ACL deficien*" ) OR TITLE-ABS-KEY ( "ACL rupture" ) OR TITLE-ABS-KEY ( "ACL tear" ) OR TITLE-ABS-KEY ( "Knee injur*" ) ) ) AND ( ( TITLE-ABS-KEY ( ( force W/5 control ) ) OR TITLE-ABS-KEY ( ( force W/5 modulation ) ) OR TITLE-ABS-KEY ( ( force W/5 fluctuation ) ) OR TITLE-ABS-KEY ( ( force W/5 variability ) ) OR TITLE-ABS-KEY ( ( force W/5 steadiness ) ) OR TITLE-ABS-KEY ( ( torque W/5 control ) ) OR TITLE-ABS-KEY ( ( torque W/5 quality ) ) OR TITLE-ABS-KEY ( ( torque W/5 variability ) ) OR TITLE-ABS-KEY ( "torque-time curve" ) OR TITLE-ABS-KEY ( ( force W/5 sense ) ) OR TITLE-ABS-KEY ( ( force W/5 perception ) ) OR TITLE-ABS-KEY ( ( force W/5 accuracy ) ) OR TITLE-ABS-KEY ( ( muscle W/0 force ) ) OR TITLE-ABS-KEY ( ( quadriceps W/5 force ) ) ) ) |
| **Embase:**  **'knee injury'**/mj OR **'anterior cruciate ligament'**/mj OR **'anterior cruciate ligament injury'**/mj OR **'anterior cruciate ligament rupture'**/mj OR **'anterior cruciate ligament reconstruction'**/mj  **'anterior cruciate ligament'**:ti,ab,kw OR **'anterior cruciate ligament reconstruction'**:ti,ab,kw OR **acl**:ti,ab,kw OR **aclr**:ti,ab,kw OR **'acl reconstruct*'**:ti,ab,kw OR **'acl injur*'**:ti,ab,kw OR **'acl deficien*'**:ti,ab,kw OR **'acl rupture'**:ti,ab,kw OR **'acl tear'**:ti,ab,kw OR **'knee injur*'**:ti,ab,kw  #1 OR #2  ((**force** NEAR/5 **control**):ti,ab,kw) OR ((**force** NEAR/5 **modulation**):ti,ab,kw) OR ((**force** NEAR/5 **fluctuation**):ti,ab,kw) OR ((**force** NEAR/5 **variability**):ti,ab,kw) OR ((**force** NEAR/5 **steadiness**):ti,ab,kw) OR ((**torque** NEAR/5 **control**):ti,ab,kw) OR ((**torque** NEAR/5 **quality**):ti,ab,kw) OR ((**torque** NEAR/5 **variability**):ti,ab,kw) OR **'torque-time curve'**:ti,ab,kw OR ((**force** NEAR/5 **sense**):ti,ab,kw) OR ((**force** NEAR/5 **perception**):ti,ab,kw) OR ((**force** NEAR/5 **accuracy**):ti,ab,kw) OR **'muscle force'**:ti,ab,kw OR ((**quadriceps** NEAR/5 **force**):ti,ab,kw)  #3 AND #4 |
| **SPORTDiscuss:**  SU ("anterior cruciate ligament" OR "anterior cruciate ligament reconstruction" OR ACL OR ACLR OR "ACL reconstruct*" OR "ACL injur*" OR "ACL deficien* OR "ACL rupture" OR "ACL tear" OR "Knee injur*")  SU ((force N5 control) OR (force N5 modulation) OR (force N5 fluctuation) OR (force N5 variability) OR (force N5 steadiness) OR (torque N5 control) OR (torque N5 quality) OR (torque N5 variability) OR “torque-time curve” OR (force N5 sense) OR (force N5 perception) OR (force N5 accuracy) OR ('muscle force") OR (quadriceps N5 force))  S1 AND S2 |
