# Supplementary material for: Changes in Quadriceps Force Control and Torque Quality Following Anterior Cruciate Ligament Injury and Reconstruction: Associations with Functional Performance—A Systematic Review and Meta-Analysis
Source: Sports Med Open. 2026 May 11;12:54. doi: 10.1186/s40798-026-00999-x (PMC13158337; doi:10.1186/s40798-026-00999-x)
Supplement: Supplementary file 2 — Supplementary Material 2. [file 40798_2026_999_MOESM2_ESM.docx]

**Sensitivity analysis for frequency content in ACL injury**

**Table S1.** Leave-one-out sensitivity analysis of quadriceps signal frequency content in ACL injury: robustness and consistency of results

| Excluded study | Combined effect size (95% CI) | p-value | I^2^ | Q | Tau^2^ | df |
| --- | --- | --- | --- | --- | --- | --- |
| Brayant et al., 2009 | 0.83 [0.43, 1.23] | <0.001 | 78.3% | 32.29 | 0.25 | 7 |
| Pua et al., 2015 | 0.86 [0.41, 1.32] | <0.001 | 78.2% | 32.09 | 0.32 | 7 |
| Tsepis et al., 2004,  90% power | 0.88 [0.45, 0.30] | <0.001 | 78.4% | 32.46 | 0.27 | 7 |
| Tsepis et al., 2004,  95% power | 0.90 [0.48, 0.31] | <0.001 | 77.6% | 31.3 | 0.26 | 7 |
| Tsepis et al., 2004,  99% power | 0.81 [0.40, 0.22] | <0.001 | 77.5% | 31.07 | 0.25 | 7 |
| Scoz et al., 2019,  100% power | 0.90 [0.49, 0.31] | <0.001 | 77.6% | 31.18 | 0.26 | 7 |
| Scoz et al., 2019,  90% power | 0.66 [0.48, 0.83] | <0.001 | 0.0% | 6.78 | <0.001 | 7 |
| Scoz et al., 2019,  95% power | 0.90 [0.49, 0.31] | <0.001 | 77.6% | 31.23 | 0.26 | 7 |
| Scoz et al., 2019,  99% power | 0.91 [0.49, 0.32] | <0.001 | 77.2% | 30.68 | 0.25 | 7 |

**Sensitivity analysis for RMSE in ACL injury**

**Table S2.** Leave-one-out sensitivity analysis of quadriceps RMSE in ACL injury: robustness and consistency of overall results

| Excluded study | Combined effect size (95% CI) | p-value | I^2^ | Q | Tau^2^ | df |
| --- | --- | --- | --- | --- | --- | --- |
| Ward et al., 2019,  Comparator: uninjured controls | 0.06 [-0.14, 0.28] | 0.542 | 0.0% | 5.66 | <0.001 | 6 |
| Ward et al., 2019,  Comparator: contralateral limb | 0.21 [-0.17, 0.59] | 0.281 | 65.0% | 17.16 | 0.17 | 6 |
| Zult et al., 2017,  Comparator: contralateral limb | 0.19 [-0.20, 0.63] | 0.338 | 65.2% | 17.22 | 0.19 | 6 |
| Zult et al., 2017, comparator: uninjured controls | 0.12 [-0.23, 0.49] | 0.488 | 59.0% | 14.64 | 0.13 | 6 |
| Zult et al., 2018,  Comparator: contralateral limb | 0.23 [-0.17, 0.64] | 0.262 | 63.7% | 16.52 | 0.19 | 6 |
| Nemati et al., 2022,  30% MVIC | 0.25 [-0.10, 0.61] | 0.164 | 61.0% | 15.40 | 0.14 | 6 |
| Nemati et al., 2022,  50% MVIC | 0.24 [-0.12, 0.61] | 0.196 | 63.0% | 16.20 | 0.15 | 6 |
| Amirshakeri et al., 2021 | 0.23 [-0.14, 0.62] | 0.220 | 63.3% | 16.35 | 0.17 | 6 |

**Sensitivity analysis for CoV in ACL injury**

**Table S3.** Leave-one-out sensitivity analysis of quadriceps CoV in ACL injury: robustness and consistency of overall results

| Excluded study | Combined effect size (95% CI) | p-value | I^2^ | Q | Tau^2^ | df |
| --- | --- | --- | --- | --- | --- | --- |
| Holman et al., 2021, 10% MVIC, uninjured controls | 0.09 [-0.11, 0.29] | 0.386 | 44.9% | 23.58 | 0.07 | 13 |
| Holman et al., 2021, 25% MVIC, uninjured controls | 0.10 [-0.10, 0.30] | 0.320 | 43% | 22.81 | 0.06 | 13 |
| Holman et al., 2021, 35% MVIC, uninjured controls | 0.07 [-0.13, 0.28] | 0.487 | 44.5% | 23.43 | 0.07 | 13 |
| Holman et al., 2021, 50% MVIC, uninjured controls | 0.07 [-0.13, 0.28] | 0.475 | 44.8% | 23.53 | 0.07 | 13 |
| Holman et al., 2021, 10% MVIC, contralateral limb | 0.09 [-0.11, 0.29] | 0.391 | 44.9% | 23.61 | 0.07 | 13 |
| Holman et al., 2021, 25% MVIC, contralateral limb | 0.10 [-0.09, 0.31] | 0.290 | 41.6% | 22.26 | 0.06 | 13 |
| Holman et al., 2021, 35% MVIC, contralateral limb | 0.06 [-0.13, 0.27] | 0.521 | 43.5% | 23.02 | 0.06 | 13 |
| Holman et al., 2021, 50% MVIC, contralateral limb | 0.07 [-0.13, 0.28] | 0.488 | 44.5% | 23.43 | 0.07 | 13 |
| Skurvydas et al., 2011 | 0.08 [-0.12, 0.28] | 0.426 | 45.2% | 23.71 | 0.07 | 13 |
| Zult et al., 2017, contralateral limb | 0.05 [-0.14, 0.25] | 0.615 | 38.9% | 21.26 | 0.05 | 13 |
| Zult et al., 2017, uninjured controls | 0.02 [-0.12, 0.16] | 0.790 | 0.0% | 10.83 | <0.001 | 13 |
| Zult et al., 2018 | 0.09 [-0.12, 0.30] | 0.396 | 44.8% | 23.56 | 0.07 | 13 |
| Nemati et al., 2022, 30%MVIC | 0.10 [-0.08, 0.30] | 0.284 | 41.0% | 22.02 | 0.05 | 13 |
| Nemati et al., 2022, 50%MVIC | 0.11 [-0.08, 0.30] | 0.264 | 39.4% | 21.45 | 0..05 | 13 |
| Lemos et al, 2024 | 0.11 [-0.09, 0.31] | 0.291 | 41.1% | 22.07 | 0.06 | 13 |

**Sensitivity analysis for frequency content in ACL reconstruction**

**Table S4.** Leave-one-out sensitivity analysis of quadriceps force signal frequency content in ACL reconstruction: robustness and consistency of overall results

| Excluded study | Combined effect size (95% CI) | p-value | I^2^ | Q | Tau^2^ | df |
| --- | --- | --- | --- | --- | --- | --- |
| Hunt et al., 2024 | 1.37 [0.84, 1.89] | <0.001 | 76.9% | 38.88 | 0.53 | 9 |
| Hunt et al., 2024 | 1.70 [1.07, 2.33] | <0.001 | 81.3% | 48.11 | 0.77 | 9 |
| Hunt et al., 2024 | 1.62 [0.97, 2.27] | <0.001 | 82.9% | 52.75 | 0.84 | 9 |
| Hunt et al., 2024 | 1.46 [0.86, 2.06] | <0.001 | 81% | 47.36 | 0.70 | 9 |
| Brayant et al., 2009 | 1.65 [0.97, 2.34] | <0.001 | 82.9% | 52.72 | 0.94 | 9 |
| Czaplicki et al., 2017 | 1.46 [0.86, 2.05] | <0.001 | 80.2% | 45.34 | 0.69 | 9 |
| Czaplicki et al., 2017 | 1.40 [0.83, 1.97] | <0.001 | 78.5% | 41.81 | 0.61 | 9 |
| Czaplicki et al., 2017 | 1.60 [0.94, 2.26] | <0.001 | 87.8% | 52.21 | 0.86 | 9 |
| Czaplicki et al., 2017 | 1.60 [0.94, 2.26] | <0.001 | 82.8% | 52.34 | 0.87 | 9 |
| Czaplicki et al., 2017 | 1.70 [1.05, 2.35] | <0.001 | 81.3% | 48.12 | 0.82 | 9 |
| Czaplicki et al., 2017 | 1.71 [1.09, 2.33] | <0.001 | 79.6% | 44.04 | 0.74 | 9 |

**Sensitivity analysis for RMSE in ACL reconstruction**

**Table S5.** Leave-one-out sensitivity analysis of quadriceps force signal RMSE in ACL reconstruction: robustness and consistency of overall results

| Excluded study | Combined effect size (95% CI) | p-value | I^2^ | Q | Tau^2^ | df |
| --- | --- | --- | --- | --- | --- | --- |
| Telianidis et al., 2014 | 0.20 [-0.05, 0.46] | 0.127 | 60.4% | 47.96 | 0.21 | 19 |
| Perraton et al., 2017 | 0.18 [-0.05, 0.41] | 0.132 | 49.4% | 37.53 | 0.14 | 19 |
| Baumeister et al., 2011, block 1 | 0.25 [-0.01, 0.52] | 0.059 | 63.8% | 52.43 | 0.23 | 19 |
| Baumeister et al., 2011, block 2 | 0.25 [-0.01, 0.52] | 0.060 | 63.8% | 52.47 | 0.23 | 19 |
| Baumeister et al., 2011, block 3 | 0.26 [-0.006, 0.53] | 0.055 | 63.6% | 52.13 | 0.23 | 19 |
| Baumeister et al., 2011, block 4 | 0.26 [-0.0004, 0.53] | 0.050 | 63.2% | 51.65 | 0.22 | 19 |
| Rice et al., 2021,  25% MVIC | 0.22 [-0.04, 0.48] | 0.108 | 63.1% | 51.51 | 0.23 | 19 |
| Rice et al., 2021,  50% MVIC | 0.20 [-0.05, 0.46] | 0.121 | 61.4% | 49.16 | 0.21 | 19 |
| Rice et al., 2021,  20-80% MVIC | 0.22 [-0.04, 0.49] | 0.107 | 63.2% | 51.62 | 0.23 | 19 |
| Sherman et al., 2022,  Contralateral limb | 0.28 [0.02, 0.54] | 0.034 | 60.4% | 48.02 | 0.21 | 19 |
| Sherman et al., 2022, uninjured controls, | 0.26 [-0.004, 0.53] | 0.054 | 62.9% | 51.22 | 0.23 | 19 |
| Sherman et al., 2023,  Contralateral limb, 100%MVIC | 0.23 [-0.03, 0.51] | 0.089 | 64.1% | 52.93 | 0.24 | 19 |
| Sherman et al., 2023,  Contralateral limb, 30% MVIC | 0.23 [-0.03, 0.50] | 0.094 | 64.0% | 52.73 | 0.24 | 19 |
| Sherman et al., 2023,  Contralateral limb, 50% MVIC | 0.25 [-0.01, 0.53] | 0.063 | 63.8% | 52.45 | 0.23 | 19 |
| Sherman et al., 2023,  Contralateral limb, 70% MVIC | 0.24 [-0.02, 0.52] | 0.074 | 64.1% | 52.95 | 0.24 | 19 |
| Sherman et al., 2023,  Uninjured controls, 100% MVIC | 0.21 [-0.05, 0.48] | 0.112 | 62.7% | 50.89 | 0.22 | 19 |
| Sherman et al., 2023,  Uninjured controls, 30% MVIC | 0.26 [-0.005, 0.53] | 0.055 | 63.3% | 51.77 | 0.23 | 19 |
| Sherman et al., 2023,  Uninjured controls, 50% MVIC | 0.27 [0.007, 0.54] | 0.044 | 62.3% | 50.44 | 0.22 | 19 |
| Sherman et al., 2023,  Uninjured controls, 70% MVIC | 0.22 [-0.04, 0.49] | 0.106 | 63.3% | 51.74 | 0.23 | 19 |
| Sherman et al., 2025,  Uninjured controls | 0.27 [0.01, 0.54] | 0.039 | 61.2% | 48.97 | 0.21 | 19 |
| Sherman et al., 2025,  Contralateral limb | 0.29 [0.03, 0.54] | 0.027 | 59.1% | 46.45 | 0.19 | 19 |

**Sensitivity analysis for CoV in ACL reconstruction**

**Table S6.** Leave-one-out sensitivity analysis of quadriceps force signal CoV in ACL reconstruction: robustness and consistency of overall results

| Excluded study | Combined effect size (95% CI) | p-value | I^2^ | Q | Tau^2^ | df |
| --- | --- | --- | --- | --- | --- | --- |
| San Martin-Mohr et al., 2018 | 0.25 [0.09, 0.41] | 0.002 | 22.3% | 29.6 | 0.03 | 23 |
| Spencer et al., 2020 | 0.21 [0.04, 0.38] | 0.013 | 31.2% | 33.44 | 0.05 | 23 |
| Goetschius and Hart, 2016 | 0.17 [0.02, 0.32] | 0.026 | 13.7% | 26.65 | 0.02 | 23 |
| Niederer et al., 2020 | 0.21 [0.04, 0.38] | 0.013 | 30.7% | 33.18 | 0.05 | 23 |
| Goetschius et al., 2015 | 0.19 [0.03, 0.35] | 0.019 | 24.9% | 30.63 | 0.04 | 23 |
| Nuccio et al, 2024, uninjured controls, 10% MVIC | 0.21 [0.05, 0.38] | 0.010 | 31.3% | 33.48 | 0.05 | 23 |
| Nuccio et al, 2024, uninjured controls, 30% MVIC | 0.21 [0.04, 0.37] | 0.012 | 30.0% | 32.88 | 0.04 | 23 |
| Nuccio et al, 2024, contralateral limb, 10% MVIC | 0.20 [0.04, 0.36] | 0.013 | 26.0% | 31.09 | 0.04 | 23 |
| Nuccio et al, 2024, contralateral limb, 30% MVIC | 0.23 [0.06, 0.40] | 0.006 | 29.6% | 32.69 | 0.04 | 23 |
| Sherman et al., 2022, contralateral limb | 0.22 [0.05, 0.39] | 0.010 | 31.5% | 33.56 | 0.05 | 23 |
| Sherman et al., 2022, uninjured controls | 0.23 [0.06, 0.40] | 0.006 | 29.5% | 32.64 | 0.04 | 23 |
| Scheurer et al., 2020, contralateral limb | 0.21 [0.04, 0.38] | 0.012 | 31% | 33.35 | 0.05 | 23 |
| Scheurer et al., 2020, uninjured controls | 0.20 [0.03, 0.36] | 0.015 | 26.9% | 31.47 | 0.04 | 23 |
| Sherman et al., 2023, contralateral limb, 100% MVIC | 0.23 [0.06, 0.39] | 0.007 | 30.7% | 33.18 | 0.05 | 23 |
| Sherman et al., 2023, contralateral limb, 30% MVIC | 0.23 [0.06, 0.39] | 0.007 | 30.7% | 33.18 | 0.05 | 23 |
| Sherman et al., 2023, contralateral limb, 50% MVIC | 0.21 [0.04, 0.37] | 0.013 | 30.4% | 33.07 | 0.05 | 23 |
| Sherman et al., 2023, contralateral limb, 70% MVIC | 0.23 [0.06, 0.39] | 0.007 | 30.7% | 33.18 | 0.05 | 23 |
| Sherman et al., 2023, uninjured controls, 100% MVIC | 0.23 [0.06, 0.40] | 0.006 | 29.6% | 32.68 | 0.04 | 23 |
| Sherman et al., 2023, uninjured controls, 30% MVIC | 0.23 [0.06, 0.39] | 0.007 | 30.7% | 33.19 | 0.05 | 23 |
| Sherman et al., 2023, uninjured controls, 50% MVIC | 0.23 [0.06, 0.39] | 0.007 | 30.7% | 33.19 | 0.05 | 23 |
| Sherman et al., 2023, uninjured controls, 70% MVIC | 0.23 [0.06, 0.39] | 0.007 | 30.7% | 33.19 | 0.05 | 23 |
| Sherman et al., 2025, uninjured controls | 0.24 [0.08, 0.40] | 0.003 | 26.0% | 31.09 | 0.04 | 23 |
| Sherman et al., 2025, contralateral limb | 0.25 [0.09, 0.41] | 0.002 | 22.5% | 29.67 | 0.03 | 23 |
| Hunt et al., 2024 | 0.22 [0.05, 0.39] | 0.008 | 31.1% | 33.40 | 0.05 | 23 |
| Jo and Kim, 2025 | 0.20 [0.04, 0.35] | 0.011 | 22.8% | 29.79 | 0.03 | 23 |
