# Supplementary figures and images for: Changes in Quadriceps Force Control and Torque Quality Following Anterior Cruciate Ligament Injury and Reconstruction: Associations with Functional Performance—A Systematic Review and Meta-Analysis
Source: Sports Med Open. 2026 May 11;12:54. doi: 10.1186/s40798-026-00999-x (PMC13158337; doi:10.1186/s40798-026-00999-x)

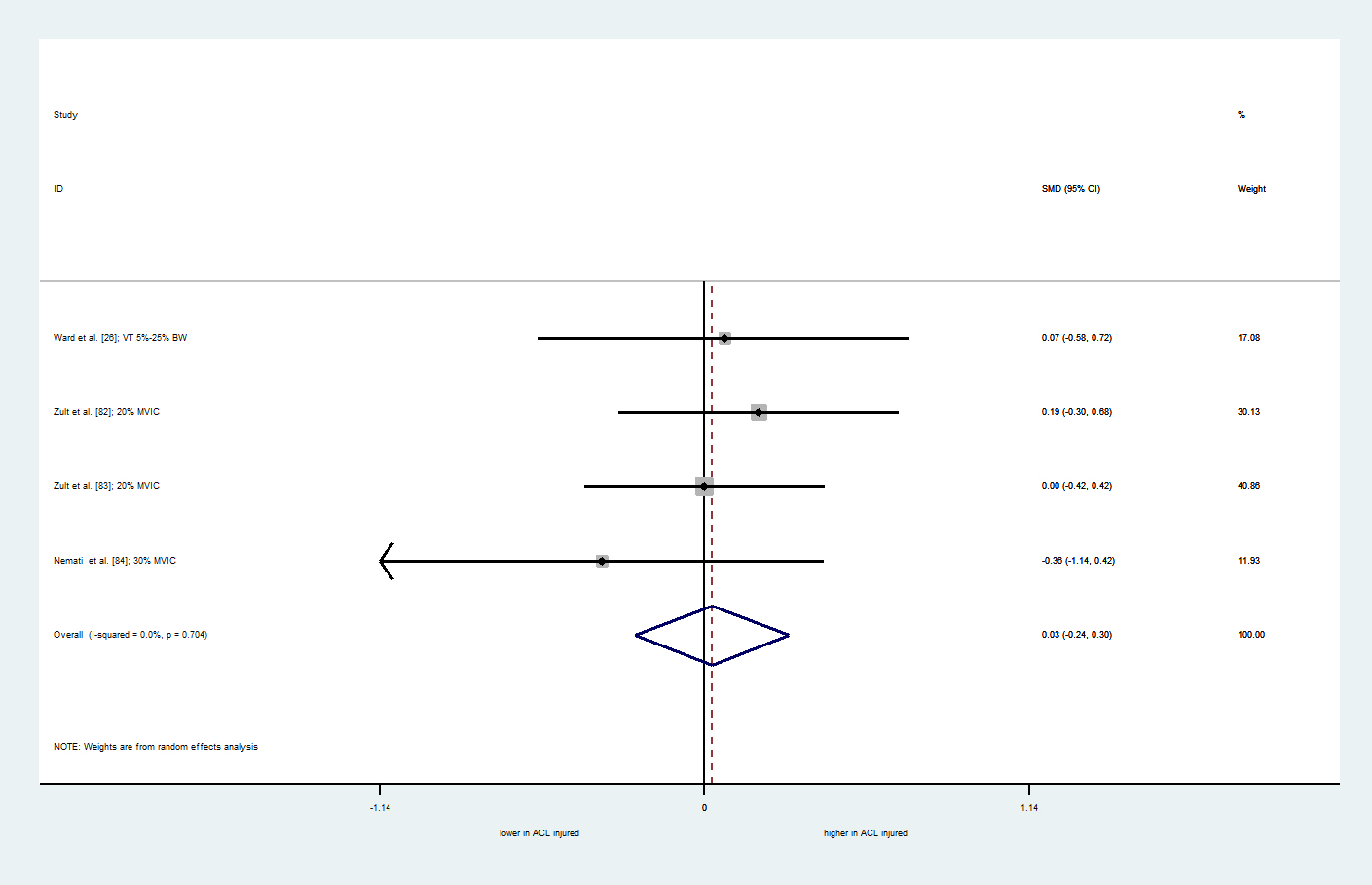

Supplement: Supplementary file 3 — Supplementary Material 3. Forest plot depicting the pooled standardized mean difference (95% confidence intervals (lower limit to upper limit)) of the Root Mean Square Error of quadriceps force signal between affected limb and unaffected limb of individuals with ACL injury, in low intensity isometric contractions (<50% MVIC). SMD= Standardized mean differences. 95%CI= 95% Confidence interval. ACL= Anterior cruciate ligament. MVIC: Maximum Voluntary Isometric Contraction. VT= Variable target. BW= Body weight. [file 40798_2026_999_MOESM3_ESM.jpg]

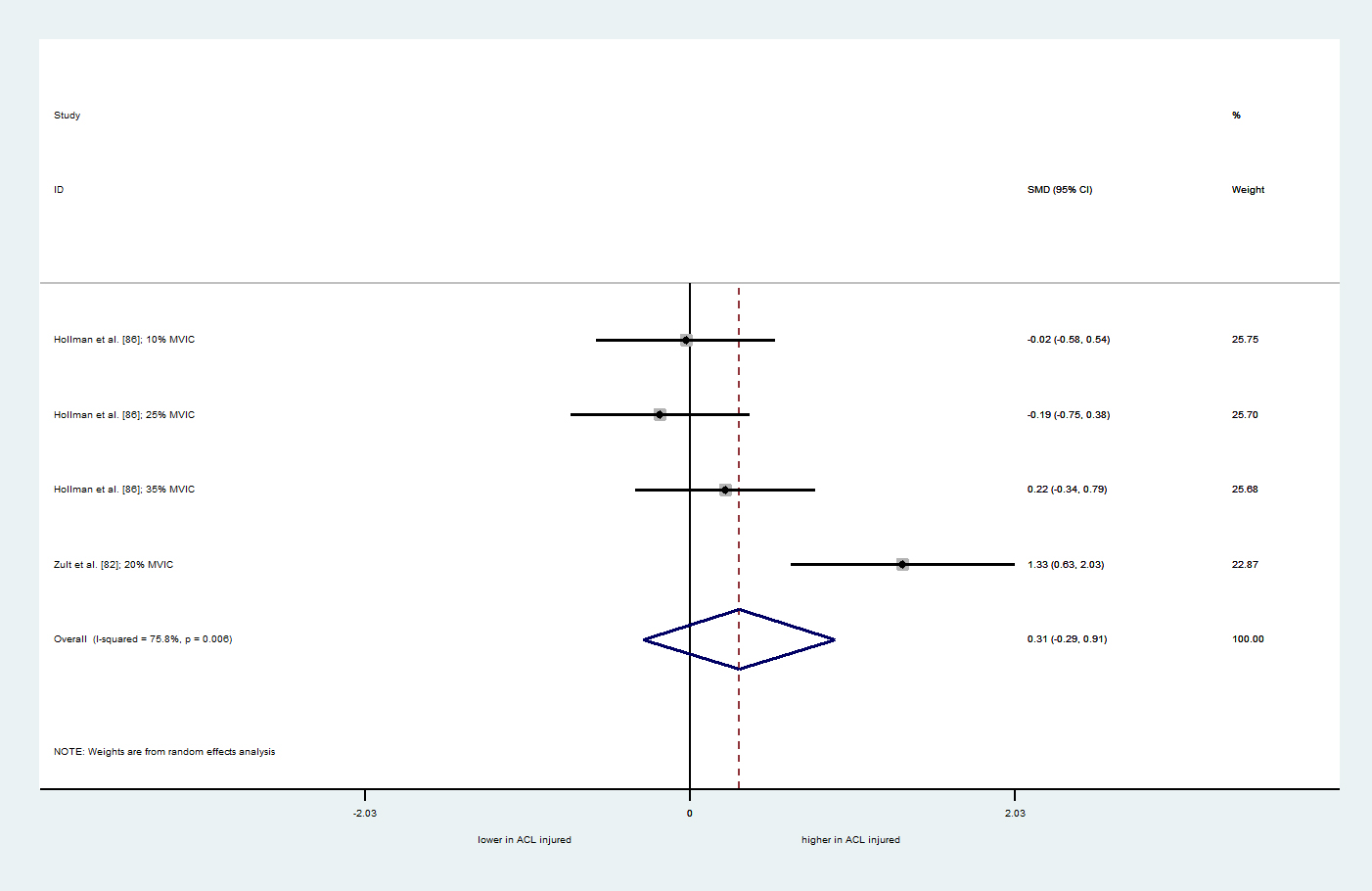

Supplement: Supplementary file 4 — Supplementary Material 4. Forest plot depicting the pooled standardized mean difference (95% confidence intervals (lower limit to upper limit)) of the Coefficient of Variation of quadriceps force signal between individuals with ACL injury and healthy controls, in low intensity isometric contractions (<50% MVIC). SMD= Standardized mean differences. 95%CI= 95% Confidence interval. ACL= Anterior cruciate ligament. MVIC: Maximum Voluntary Isometric Contraction. [file 40798_2026_999_MOESM4_ESM.jpg]

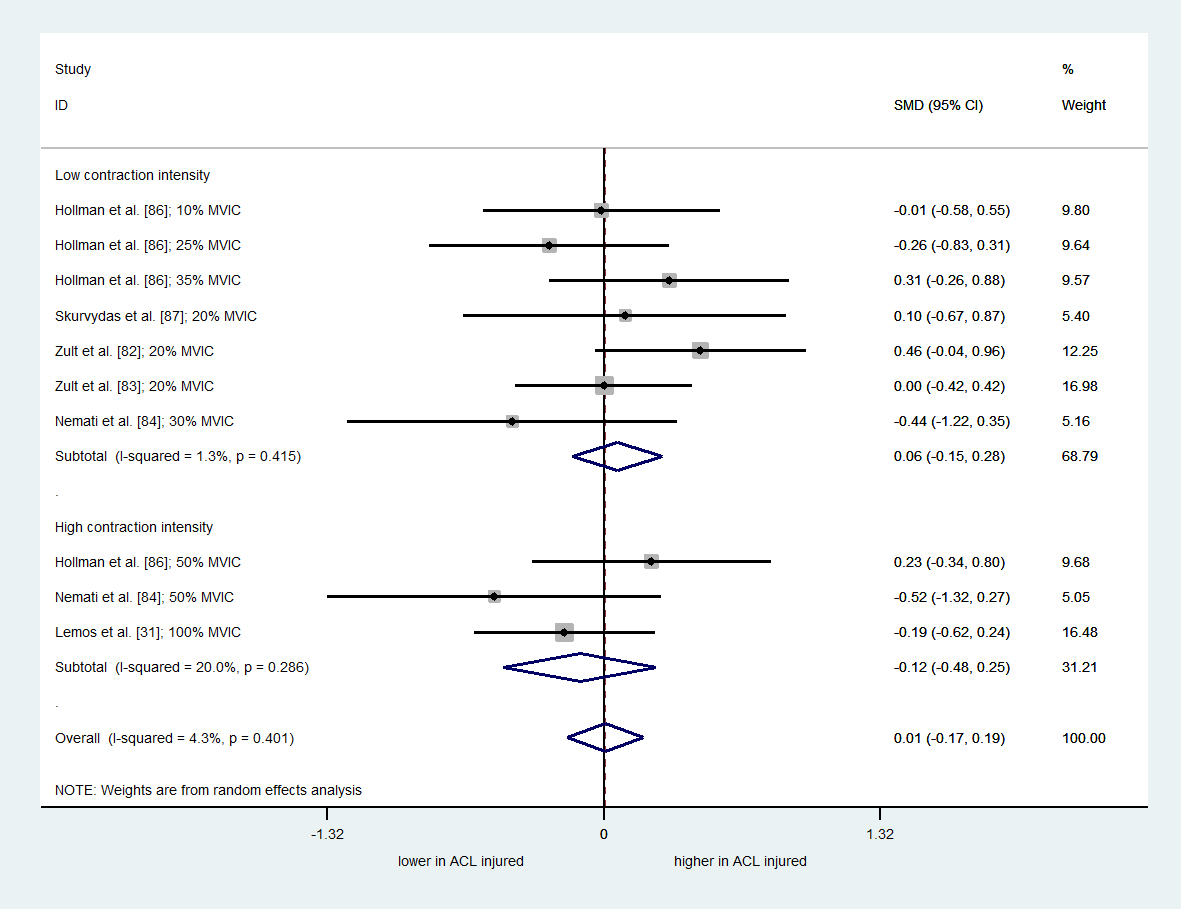

Supplement: Supplementary file 5 — Supplementary Material 5. Forest plot depicting the pooled standardized mean difference (95% confidence intervals (lower limit to upper limit)) of the Coefficient of Variation of quadriceps force signal between affected limb and unaffected limb of individuals with ACL injury, in 1: low (<50% MVIC) and 2: high (≥50% MVIC) intensity isometric contractions. SMD= Standardized mean differences. 95%CI= 95% Confidence interval. ACL= Anterior cruciate ligament. MVIC: Maximum Voluntary Isometric Contraction. [file 40798_2026_999_MOESM5_ESM.jpg]

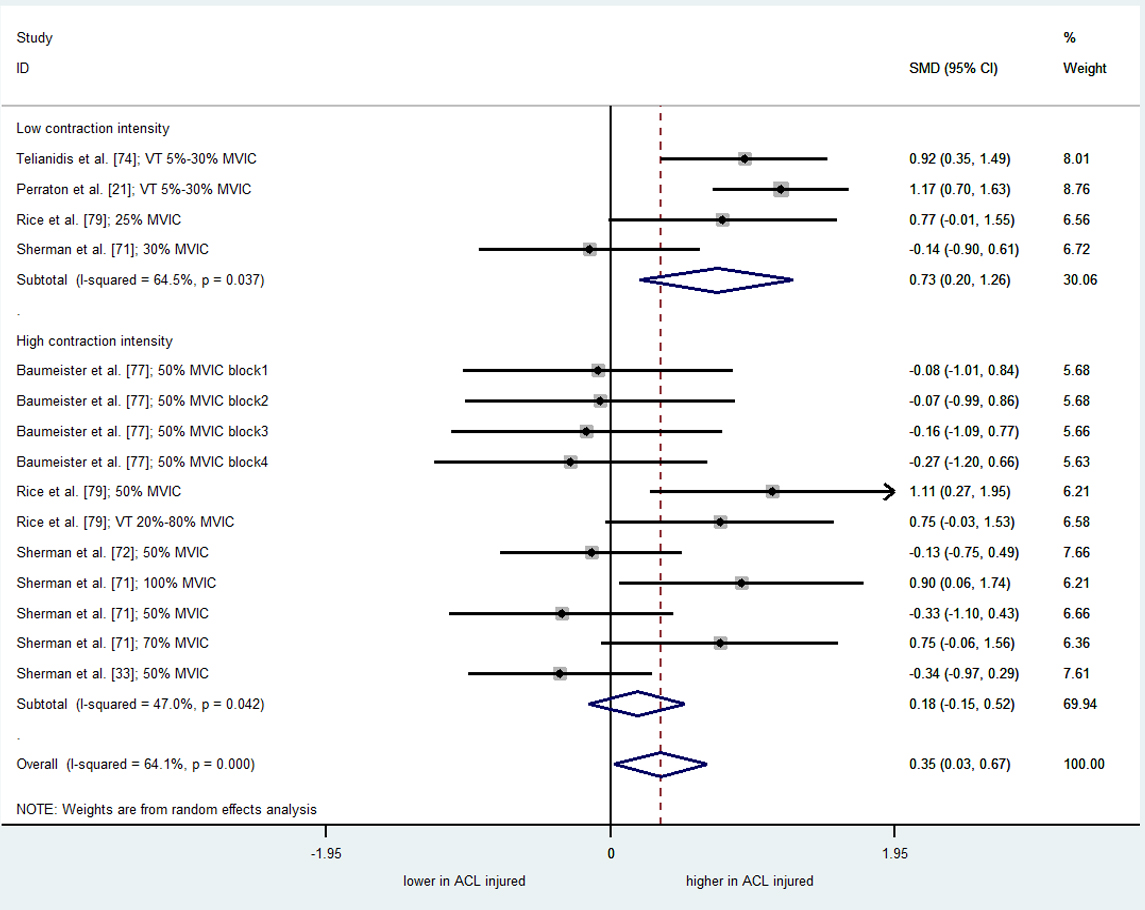

Supplement: Supplementary file 6 — Supplementary Material 6. Forest plot depicting the pooled standardized mean difference (95% confidence intervals (lower limit to upper limit)) of the Root Mean Square Error of quadriceps force signal between individuals with ACL reconstruction and healthy controls, in 1: low (<50% MVIC) and 2: high (≥50% MVIC) intensity isometric contractions. SMD= Standardized mean differences. 95%CI= 95% Confidence interval. ACL= Anterior cruciate ligament. MVIC: Maximum Voluntary Isometric Contraction. [file 40798_2026_999_MOESM6_ESM.jpg]

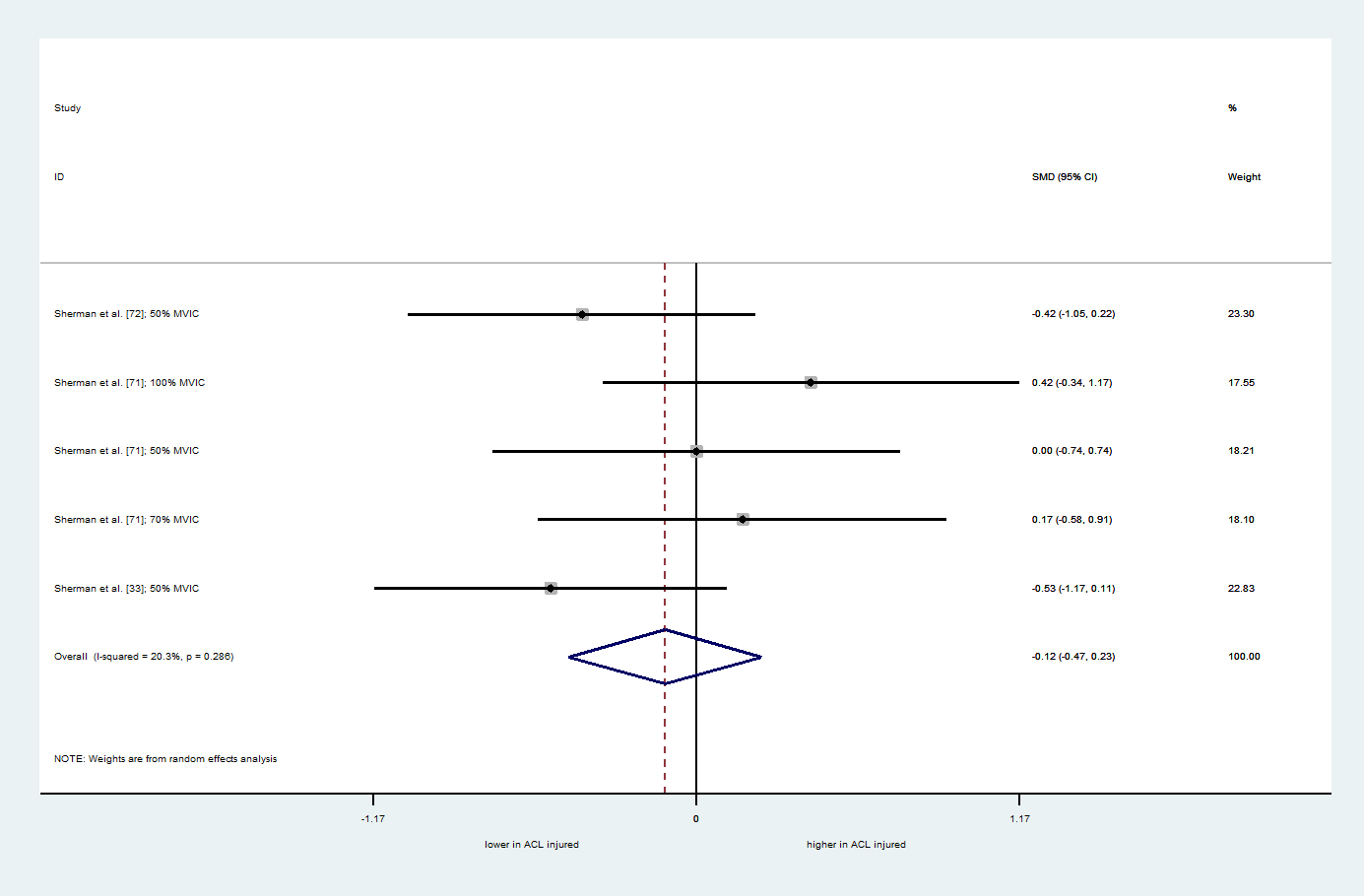

Supplement: Supplementary file 7 — Supplementary Material 7. Forest plot depicting the pooled standardized mean difference (95% confidence intervals (lower limit to upper limit)) of the Root Mean Square Error of quadriceps force signal between affected limb and unaffected limb of individuals with ACL reconstruction, in high (≥50% MVIC) intensity isometric contractions. SMD= Standardized mean differences. 95%CI= 95% Confidence interval. ACL= Anterior cruciate ligament. MVIC: Maximum Voluntary Isometric Contraction. [file 40798_2026_999_MOESM7_ESM.jpg]

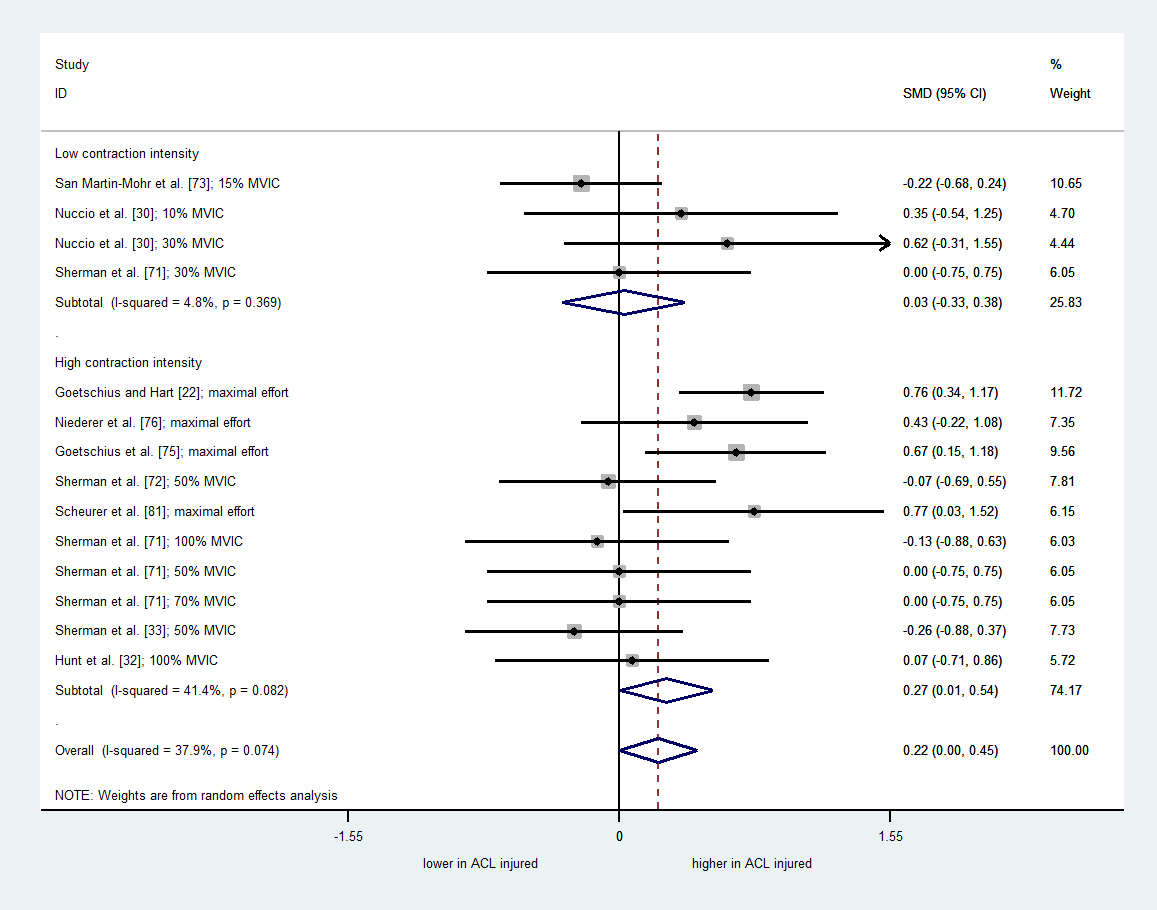

Supplement: Supplementary file 8 — Supplementary Material 8. Forest plot depicting the pooled standardized mean difference (95% confidence intervals (lower limit to upper limit)) of the Coefficient of Variation of quadriceps force signal between individuals with ACL reconstruction and healthy controls, in 1: low (<50% MVIC) and 2: high (≥50% MVIC) intensity isometric contractions. SMD= Standardized mean differences. 95%CI= 95% Confidence interval. ACL= Anterior cruciate ligament. MVIC: Maximum Voluntary Isometric Contraction. [file 40798_2026_999_MOESM8_ESM.jpg]

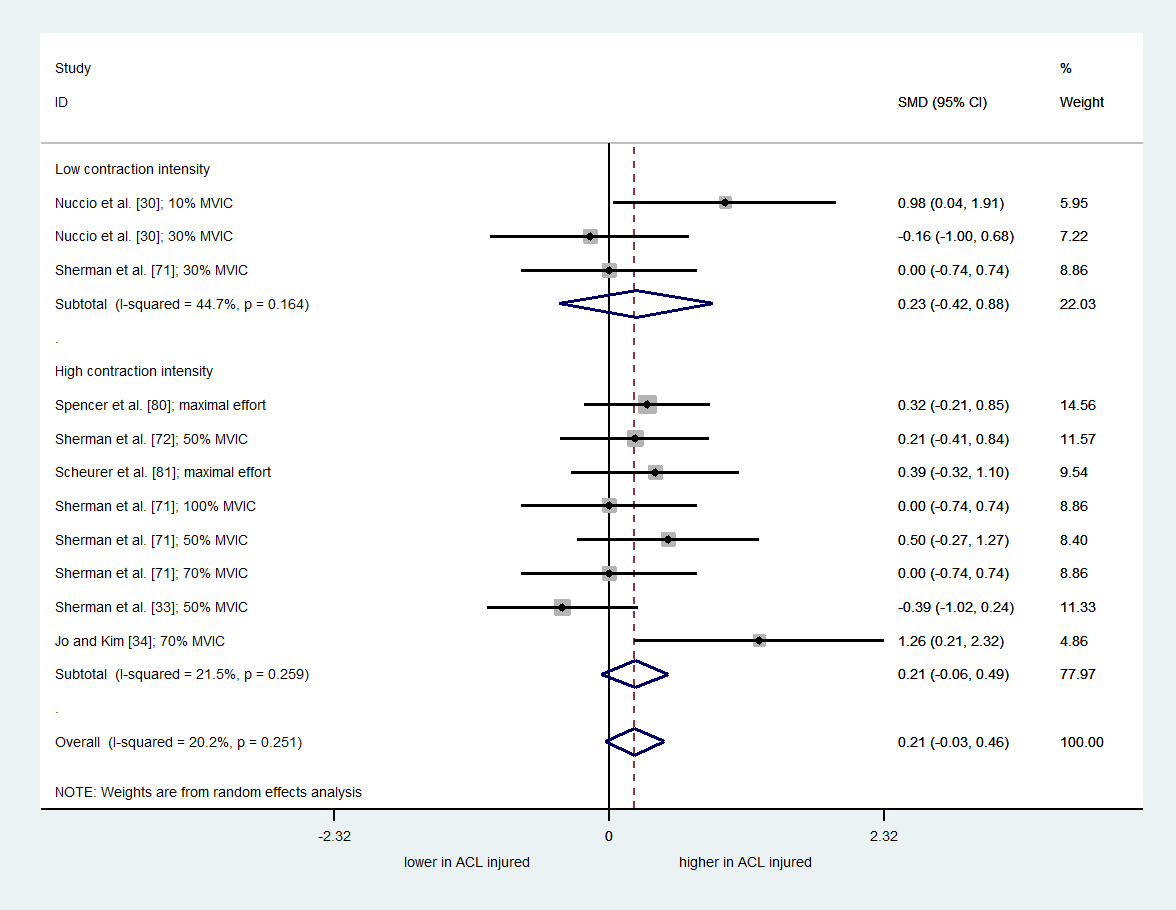

Supplement: Supplementary file 9 — Supplementary Material 9. Forest plot depicting the pooled standardized mean difference (95% confidence intervals (lower limit to upper limit)) of the Coefficient of Variation of quadriceps force signal between affected limb and unaffected limb of individuals with ACL reconstruction, in 1: low (<50% MVIC) and 2: high (≥50% MVIC) intensity isometric contractions. SMD= Standardized mean differences. 95%CI= 95% Confidence interval. ACL= Anterior cruciate ligament. MVIC: Maximum Voluntary Isometric Contraction. [file 40798_2026_999_MOESM9_ESM.jpg]
